# *Bcar1*/p130Cas is essential for ventricular development and neural crest cell remodelling of the cardiac outflow tract

**DOI:** 10.1093/cvr/cvab242

**Published:** 2021-07-16

**Authors:** Marwa Mahmoud, Ian Evans, Laura Wisniewski, Yuen Tam, Claire Walsh, Simon Walker-Samuel, Paul Frankel, Peter Scambler, Ian Zachary

**Affiliations:** Centre for Cardiometabolic and Vascular Science, BHF Laboratories, UCL Division of Medicine, 5 University Street, London WC1E 6JF, UK; Centre for Cardiometabolic and Vascular Science, BHF Laboratories, UCL Division of Medicine, 5 University Street, London WC1E 6JF, UK; Centre for Cardiometabolic and Vascular Science, BHF Laboratories, UCL Division of Medicine, 5 University Street, London WC1E 6JF, UK; Centre for Cardiometabolic and Vascular Science, BHF Laboratories, UCL Division of Medicine, 5 University Street, London WC1E 6JF, UK; UCL Centre for Advanced Biomedical Imaging, Paul O'Gorman Building, 72 Huntley Street, London WC1E 6DD, UK; UCL Centre for Advanced Biomedical Imaging, Paul O'Gorman Building, 72 Huntley Street, London WC1E 6DD, UK; Institute of Cardiovascular Science, University College London, 5 University Street, London WC1E 6JF, UK; Developmental Biology of Birth Defects Section, UCL Institute of Child Health, 30 Guilford Street, London WC1N 1EH, UK; Centre for Cardiometabolic and Vascular Science, BHF Laboratories, UCL Division of Medicine, 5 University Street, London WC1E 6JF, UK

**Keywords:** Bcar1, Outflow tract septation, Epithelial-to-mesenchymal transformation, Heart development

## Abstract

**Aims:**

The adapter protein p130Cas, encoded by the *Bcar1* gene, is a key regulator of cell movement, adhesion, and cell cycle control in diverse cell types. *Bcar1* constitutive knockout mice are embryonic lethal by embryonic days (E) 11.5–12.5, but the role of *Bcar1* in embryonic development remains unclear. Here, we investigated the role of *Bcar1* specifically in cardiovascular development and defined the cellular and molecular mechanisms disrupted following targeted *Bcar1* deletions.

**Methods and results:**

We crossed *Bcar1* floxed mice with Cre transgenic lines allowing for cell-specific knockout either in smooth muscle and early cardiac tissues (*SM22-Cre*), mature smooth muscle cells (*smMHC-Cre*), endothelial cells (*Tie2-Cre*), second heart field cells (*Mef2c-Cre*), or neural crest cells (NCC) (*Pax3-Cre*) and characterized these conditional knock outs using a combination of histological and molecular biology techniques. Conditional knockout of *Bcar1* in *SM22*-expressing smooth muscle cells and cardiac tissues (*Bcar1^SM22KO^*) was embryonically lethal from E14.5–15.5 due to severe cardiovascular defects, including abnormal ventricular development and failure of outflow tract (OFT) septation leading to a single outflow vessel reminiscent of persistent truncus arteriosus. *SM22*-restricted loss of *Bcar1* was associated with failure of OFT cushion cells to undergo differentiation to septal mesenchymal cells positive for SMC-specific α-actin, and disrupted expression of proteins and transcription factors involved in epithelial-to-mesenchymal transformation (EMT). Furthermore, knockout of *Bcar1* specifically in NCC (*Bcar1^PAX3KO^*) recapitulated part of the OFT septation and aortic sac defects seen in the *Bcar1^SM22KO^* mutants, indicating a cell-specific requirement for *Bcar1* in NCC essential for OFT septation. In contrast, conditional knockouts of *Bcar1* in differentiated smooth muscle, endothelial cells, and second heart field cells survived to term and were phenotypically normal at birth and postnatally.

**Conclusion:**

Our work reveals a cell-specific requirement for *Bcar1* in NCC, early myogenic and cardiac cells, essential for OFT septation, myocardialization and EMT/cell cycle regulation and differentiation to myogenic lineages.

## Graphical Abstract

**Graphical Abstract:**
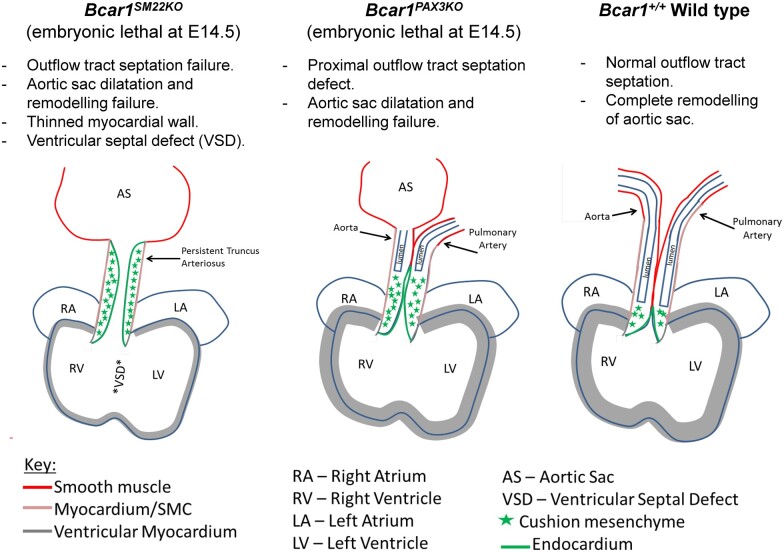

## 1. Introduction

The adapter protein p130Cas, encoded by the *Bcar1* gene, plays a critical role in chemotactic signalling in diverse cell types, through regulation of the actin cytoskeleton and its function in multimolecular complexes of focal adhesions.^1,^^2^ Tyrosine phosphorylation of Bcar1/p130Cas at multiple sites in its substrate domain is thought to play a key role in mediating protein–protein interactions, downstream effector activation, signalling and cell migration in response to cytokines, and growth factors.^1–7^ However, information about the *in vivo* role of *Bcar1*/p130Cas is very limited. *Bcar1*-null embryos died *in utero* between embryonic days (E) 11.5–12.5, exhibiting severe defects in development of the heart and prominent dilation of major blood vessels.^8^ These findings showed that *Bcar1*/p130Cas is essential for normal embryonic development, but did not indicate whether defects observed in *Bcar1*-null embryos arose from a specific role of *Bcar1*/p130Cas in cardiovascular development or were an indirect effect of complete organismal loss of *Bcar1*, and provided no indication of the molecular or cellular mechanisms involved. Mutant mice with a global deletion of the *Bcar1*/p130Cas exon 2-encoded SH3 domain died *in utero* at E12.5–E13.5, with severe liver degeneration, but display no defects in development of the heart or other major organs.^9^ Furthermore, other conditional tissue-specific ablations of *Bcar1*/p130Cas all produced live viable progeny and did not cause developmental defects.^10–12^ Thus far, studies of conditional knock outs for *Bcar1* do not account for the embryonic lethality displayed by *Bcar1*-null mice.

Division of the common outflow tract (OFT) into the pulmonary and aortic trunks, is a fundamental process in development of the mammalian heart, occurring in the mouse embryo from E11.5–E15.^13^ OFT septation into the pulmonary and aortic trunks occurs progressively in a distal to proximal order towards the ventricles and involves the coordinated migration of progenitors, including cardiac neural crest cells (CNCC),^14^ smooth muscle progenitor cells, and myocardial cells of the second heart field.^15^ Initially the aortic sac (AS) is invaded by progenitors, then the distal portion of the OFT (the truncus) is divided by the aortico-pulmonary septum, which is purely neural crest cells (NCC) in origin, followed by septation of the proximal OFT (the conus) by fusion of the OFT cushions (OTCs) (also called the bulbar/conotruncal ridges). Essential to septation of the proximal OFT is the differentiation of progenitors into mesenchymal and myocardial cells, resulting from the endocardial epithelial-to-mesenchymal transformation (EMT).^13–15^

The role of *Bcar1*/p130Cas in cardiovascular development was investigated herein by generating conditional cell type-specific deletions of *Bcar1* targeting the major cell types in the developing cardiovascular system. 0Mice with conditional knock out of *Bcar1*/p130Cas in smooth muscle cells (SMC) (*Bcar1^fl/fl^* cross with *smMHC-Cre* mice) or endothelial cells (*Tie2-Cre*) were phenotypically normal. In contrast, deletion of *Bcar1* in early smooth muscle and cardiac cells using *SM22-Cre* mice caused severe defects in cardiovascular development from E11.5, including severely impaired OFT septation, aberrant remodelling of the ventricular myocardium, ventricular septal defects, and a grossly dilated AS. Furthermore, NCC-specific knockout, using the *Pax3-Cre* transgenic line,^16^ replicated key features of the *Bcar1^SM22KO^* mutant phenotype, including defective OFT septation and an abnormally dilated AS, indicating a specific role for *Bcar1* in NCC essential for OFT septation and remodelling of the AS. *SM22*- and *Pax3*-restricted loss of *Bcar1* was also associated with disruption of mechanisms important for myogenic differentiation and EMT. These findings demonstrate an essential role for *Bcar1* in migration and differentiation of cells required for OFT septation and development of the ventricular myocardium.

## 2. Materials and methods

### 2.1 Generation and genotyping of *Bcar1* conditional knockout mice

*Bcar1^fl/fl^* mice were generated by flanking exons 7 and 8 of the *Bcar1* gene by *loxP* sites to allow their removal upon Cre-mediated action. Exons 7 and 8, encode most of the C-terminal *Bcar1*/p130Cas region and the 3′UTR, containing the endogenous polyadenylation site. The recombined embryonic stem cells were injected into blastocysts derived from an albino C57BL/6 strain (C57BL/6J-Tyrc-2J/J) to produce chimaeras (genOway, Lyon, France). Male chimaeras were subsequently bred with C57BL/6 Flp-deleter females to generate the F1 generation of *Bcar1^fl/fl^* mice (Supplementary material online, *Figure S1*). Targeted deletion of *Bcar1* was achieved by crossing female *Bcar1^fl/fl^* mice to either heterozygous male *smMHC-Cre*,^17^*SM^22^-Cre*,^18^*Tie^2^-Cre*,^19^*Mef2c-Cre*,^20^*or Pax^3^-Cre*^16^ mice to produce *Bcar1* knockouts in, respectively, SMC (*Bcar1^fl/fl^*; *smMHC-Cre^+/^*^*−*^, hereafter *Bcar1^SMKO^*), SMC and cardiac tissues (*Bcar1^fl/fl^*; *SM22-Cre^+/^*^*−*^; hereafter *Bcar1^SM22KO^*), endothelial cells (*Bcar1^fl/fl^*; *Tie2-Cre^+/^*^*−*^; hereafter *Bcar1^TIE2KO^*), second heart field cells (*Bcar1^fl/fl^*; *Mef2c-Cre^+/^*^*−*^; hereafter *Bcar1^MEF2CKO^*), or NCC (*Bcar1^fl/fl^*; *Pax3-Cre^+/^*^*−*^; hereafter *Bcar1^PAX3KO^*). Some lines were crossed to a Rosa26 reporter strain to allow detection of Cre activity by X-gal staining.^21^ Mice were backcrossed to the C57BL/6 background for at least five generations, and all mutants were compared with wild-type littermates. Genotyping of genomic DNA extracted from ear biopsies (adult mice) or tail tips (neonates and embryos) was performed by polymerase chain reaction using the primers and conditions listed in Supplementary material online, *Table S1*.

#### 2.1.1 Ethics statement

No anaesthetic/analgesic agents were used. Euthanasia was performed by overexposure to CO_2_ gas in a closed chamber followed by cervical dislocation. Animal maintenance, husbandry, and procedures conform to the guidelines from Directive 2010/63/EU of the European Parliament and were conducted in accordance with the Animal Care and Ethics Guidelines of University College London (UK) and the United Kingdom Home Office Animals (Scientific Procedures) Act of 1986.

### 2.2 Western blotting

Aortic tissue from adult *Bcar1^SMKO^* mice, or right ventricle (RV) and OFT tissue from *Bcar1^SM22KO^* embryos was homogenized in RIPA lysis buffer supplemented with protease inhibitors (Sigma #P2714). Equivalent amounts of protein were blotted with the primary antibodies listed in Supplementary material online, *Table S2*. Protein bands were visualized with horseradish peroxidase-conjugated secondary antibodies (Santa Cruz) and ECL 2 Western Blotting Substrate kit (Thermo Scientific) according to the manufacturers’ instructions.

### 2.3 X-Gal staining

Tissues were stained for β-galactosidase activity using a kit from InvivoGen (catalog #rep-lz-t), according to the protocol provided. Briefly, embryos were dissected, fixed with 0.5% glutaraldehyde in phosphate buffered saline (PBS)/MgCl_2_ solution on ice, and then stained with X-Gal staining solution overnight at 37°C followed by extensive washes with PBS. Samples were then embedded in paraffin wax, and 7 μm sections were cut and counterstained with Nuclear Fast Red.

### 2.4 Histological and immunohistochemical staining

Embryos were dissected at the specified ages and fixed with 1× zinc fixative (BD Biosciences #552658) overnight at room temperature. Samples were then processed to paraffin and embryos were embedded on their backs to provide sections in the frontal orientation, which offers a good view of the vessels entering and leaving the heart, and also gives a clear view of OFT septation. Serial tissue sections, 7 μm thickness, were either stained with haematoxylin and eosin (H&E) or subjected to immunohistochemistry/immunofluorescence with primary and secondary antibodies (Supplementary material online, *Table S3*).

### 2.5 Ink injections

Ink injections were performed on embryos, which had been fixed in 4% paraformaldehyde overnight. The OFT was injected with Indian ink (Pelican) diluted 1:5 in PBS using a microinjection glass capillary needle. Images were acquired with a camera attached to a Leica stereo microscope.

### 2.6 Amira reconstructions and HREM imaging

The 7 μm-thick serial sections from E14.5 control, *Bcar1^SM22KO^*, and *Bcar1^PAX3KO^* embryos were stained with H&E and scanned with the NanoZoomer HT slide scanner (Hamamatsu Photonics Ltd.). Image files were uploaded to the Amira software where they were aligned and processed to produce animations illustrating septation of the entire OFT. For High Resolution Episcopic Microscopy (HREM) analysis, embryos were fixed in 4% PFA overnight, dehydrated through an acetone series (50%, 80%, 100%, 100%, and 100%), then infiltrated with Technovit 8100 resin (Taab Laboratories) through a series of 50:50 (Technovit 8100: Acetone) for 24 h, 75:25 (Technovit 8100: acetone) for 24 h, and 100% Technovit 8100 for a final 24 h. All solutions had the addition of 1 mg/ml Eosin B (Sigma) and were performed at 4°C with constant agitation. Finally, embryos were embedded in Technovit 8100 under vacuum at 4°C. After 48 h, samples were imaged using the Optical HREM system (Indigo Scientific) using 405/495 nm Ex/Em filter set, at appropriate resolution to ensure the entire embryo was in the field of view, and using a slice thickness to provide approximately isotropic resolution. Image segmentation was performed by manual annotation. Both segmentation and volume rendering were performed with Amira and ImageJ software.

### 2.7 RNASeq analysis

Whole RNA was extracted from dissected OFTs from E11.5 *Bcar1^SM22KO^* mutants and littermate controls using the QIAGEN RNeasy Micro Kit, according to the manufacturers’ instructions. Samples were processed for RNASeq analysis by the UCL Genomics facility using the NextSeq. Differential expression analysis was performed using Galaxy software with the local fit parametric. Raw *P*-values were corrected using the Benjamini–Hochberg method to account for multiple testing.

### 2.8 Statistical analysis

Data are presented as means ± SEM. Statistical significance was determined using the 2-tailed Student’s *t*-test and *P* ≤ 0.05 was considered statistically significant. When quantifying the pharyngeal arch artery (PAA) diameter, the diameter across the widest part of the vessel was taken, *n* = 3 embryos were used for each genotype and at least six measurements taken from each embryo.

## 3. Results

### 3.1 *Bcar1* is essential for embryonic heart development but is not required in smooth muscle cells or endothelial cells

To investigate the role of *Bcar1* in early cardiovascular development, we generated a targeted deletion of *Bcar1* in smooth muscle and cardiac cells by crossing *Bcar1^fl/fl^* mice with the *SM22-Cr*e mouse line (Supplementary material online, *Figures S1–S3*). *SM22α* is transiently expressed in the presumptive RV (bulbus cordis) and OFT myocardium of the mouse heart between E8.0 and E12.5,^22–24^ and in vascular smooth muscle cells (VSMC) from E9.5. *SM22-Cre* promoter activity was detected in the heart and OFT at E8.5–E9 as determined by mapping β-galactosidase expression in *Bcar1^SM22KO^* mice crossed to a Rosa26 reporter strain (Supplementary material online, *Figure S2A*). Recombination of the floxed *Bcar1* allele was confirmed by genotyping (Supplementary material online, *Figure S2B*), and immunoblotting of cardiac tissue from *Bcar1^SM22KO^* mice including the RV and OFT verified that excision of the conditional *Bcar1* allele resulted in depletion of the *Bcar1*-encoded p130Cas protein compared with wild-type littermates (Supplementary material online, *Figure S2C*). To distinguish possible functions of *Bcar1* in cardiomyocytes and SMC, we generated mice with conditional *Bcar1* knock out restricted to SMC by crossing *Bcar1^fl/fl^* with *smMHC-Cre* mice to produce the *Bcar1^SMKO^* line (Supplementary material online, *Figure S2D–F*). SMC-specific Myosin Heavy Chain (smMHC or Myh11) is a highly specific marker of smooth muscle, detected in the early aorta (Ao) from E10.5, in aortic arch arteries from E12.5, and in the immature coronary artery plexuses from E14.5.^25^ SMC-specific loss of *Bcar1* was verified by SMC-restricted β-galactosidase expression in the heart, the presence of the recombined allele, and by SMC-specific loss of *Bcar1*/p130Cas protein in *Bcar1^SMKO^* mice as assessed by immunoblot of aortic tissue (Supplementary material online, *Figure S2D–F*). We also assessed the developmental role of *Bcar1* in endothelial cells by generating mice with *Tie2*-restricted *Bcar1* deletion (*Bcar1^TIE2KO^*).

*Bcar1^SM22KO^* mutants, in which *Bcar1* was ablated in both SMCs and early cardiac tissues, were embryonic lethal by E14.5–E15.5, and by E14.5 ∼50% of *Bcar1^SM22K^*^O^ embryos were starting to be reabsorbed while the remainder showed signs of severe morbidity including evidence of haemorrhaging and severely abnormal heart morphology (Supplementary material online, *Figure S3A and B*). This phenotype was fully penetrant and no live *Bcar1^SM22KO^* progeny was produced (Supplementary material online, *Figure S3A*). In contrast, *Bcar1^SMKO^* and *Bcar1^TIE2KO^* progeny were healthy, viable, and born at the expected Mendelian ratios (Supplementary material online, *Figure S3C*), with no overt phenotype, nor detectable peri- or early post-natal mortality. Histological examination of the vasculature of the heart and lungs of neonatal *Bcar1^SMKO^* mice (9 days old) also revealed no apparent vessel abnormalities (Supplementary material online, *Figure S3D*).

### 3.2 *Bcar1* expression in the heart and OFT

Cardiovascular Bcar1/p130Cas expression was previously reported at E11.5–E12.5,^8^ but the detailed pattern of embryonic Bcar1/p130Cas expression is unclear. To gain insight into regions of the developing heart, where *Bcar1*/p130Cas is most prominent, the expression pattern of Bcar1/p130Cas in the heart and large vessels was examined by immunostaining from E10.5 up to E13.5. *Bcar1*/p130Cas protein expression was not apparent in the myocardium or OFT at E10.5 (Supplementary material online, *Figure S4A and B*), and was first detected at E11.5 in the cushion mesenchyme of the OFT, developing ventricular septum and endocardium (Supplementary material online, *Figure S4C, D and I*), expression becoming more pronounced in the OTC mesenchyme by E12.5. *Bcar1*/p130Cas was also expressed strongly in the epicardium, endocardium, and more weakly in the cardiomyocytes at E12.5 (Supplementary material online, *Figure S4E, F and J*). By E13.5 *Bcar1*/p130Cas expression was no longer seen in the OFT septum but was localized to the aortic and pulmonary valve leaflets and remained strongly expressed in the epicardium (Supplementary material online, *Figure S4G and H*).

### 3.3 *Bcar1^SM22KO^* mice exhibit severe defects in the remodelling of the ventricular myocardium, and OFT septation

**Figure 1:**
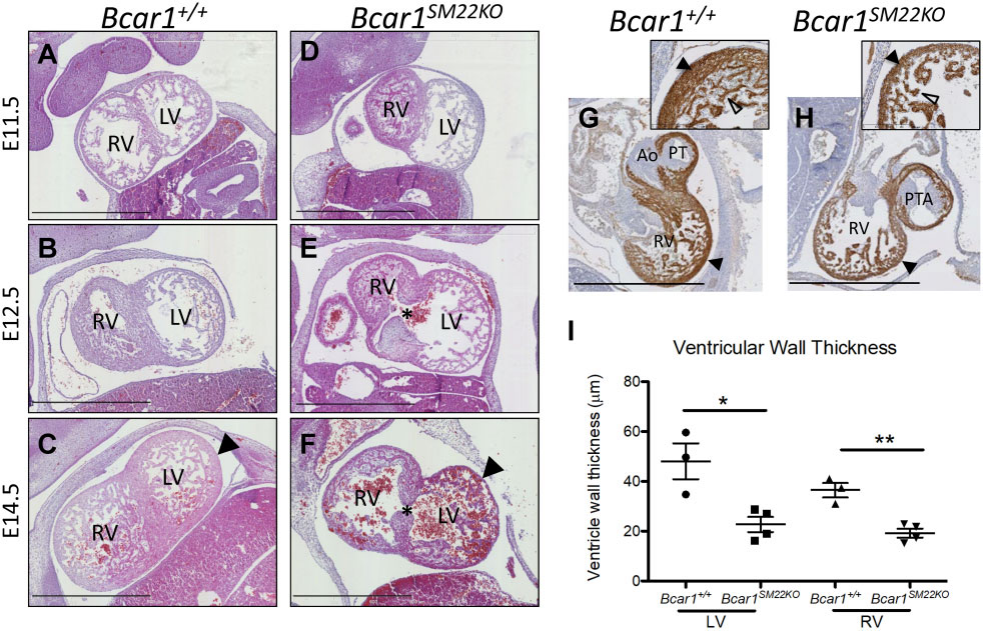
Ventricular defects in *Bcar1^SM22KO^* embryos. Ventricular development in control (*A*–*C* and *G*) and *Bcar1^SM22KO^* embryos (*D*–*F* and *H*). At E11.5 *Bcar1^SM22KO^* embryos (*D*) had an under-developed RV compared to control littermates (*A*). By E14.5 *Bcar1^SM22KO^* embryos (*F*) exhibited defects in the remodelling of the ventricular myocardium, which included thinning of the myocardial wall (arrowhead) and fewer ventricular trabeculations compared to littermate controls (*C*). An interventricular septal defect was seen in the mutants at E12.5 and E14.5 (asterisk in *E* and *F*) compared with littermate controls (*B* and *C*). MF20 staining, which labels the cardiac myosin heavy chain, indicates thinning of the ventricular myocardium (arrowheads) in E14.5 *Bcar1^SM22KO^* embryos (*H*) compared to littermate controls (*G*); higher magnification views are shown in boxed regions, arrowheads indicate the ventricular wall and hollow arrowheads highlight the ventricular trabeculations. (*I*) Quantification of LV and RV wall thickness in *Bcar1^+/+^* (*n*=3, white bar) and *Bcar1^SM22KO^* mice (*n*=4, black bar); *P=0.0155 and **P=0.0032 as determined by 2-tailed *t*-test. Scale bars are 900 μm. Ao, aorta; PT, pulmonary trunk; PTA, persistent truncus arteriosus.

The underlying causes of the embryonic lethality of the *Bcar1^SM22KO^* mice were investigated by histological analysis of the embryonic heart. From E11.5 *Bcar1^SM22KO^* mice exhibited defects in ventricular development, particularly the RV (*Figure 1A and D*). A ventricular septal defect was also observed in the *Bcar1^SM22KO^* embryos (*Figure 1B and E*). By E14.5, *Bcar1^SM22KO^* embryos had defects in the remodelling of both the right and left ventricular myocardium, including fewer trabeculations compared to littermate controls and a significantly thinner myocardial wall (*Figure 1C, F, G, H and I*). In addition, ventricular septation, compaction of the ventricular myocardium, and formation of the ‘spongy layer’ between the compact and trabeculated ventricular tissue, which normally occur between E13 and E14,^26^ were all impaired in *Bcar1^SM22KO^* mutants (*Figure 1E and F*). Defects in trabeculation have been associated with ventricular compaction defects,^27^ which may account for the thinned myocardial walls seen in the *Bcar1^SM22KO^* mutants. Ventricular defects in *Bcar1^SM22KO^* embryos were not associated with observable effects on either cardiomyocyte proliferation or apoptosis at E13.5 (Supplementary material online, *Figure S5*).

**Figure 2:**
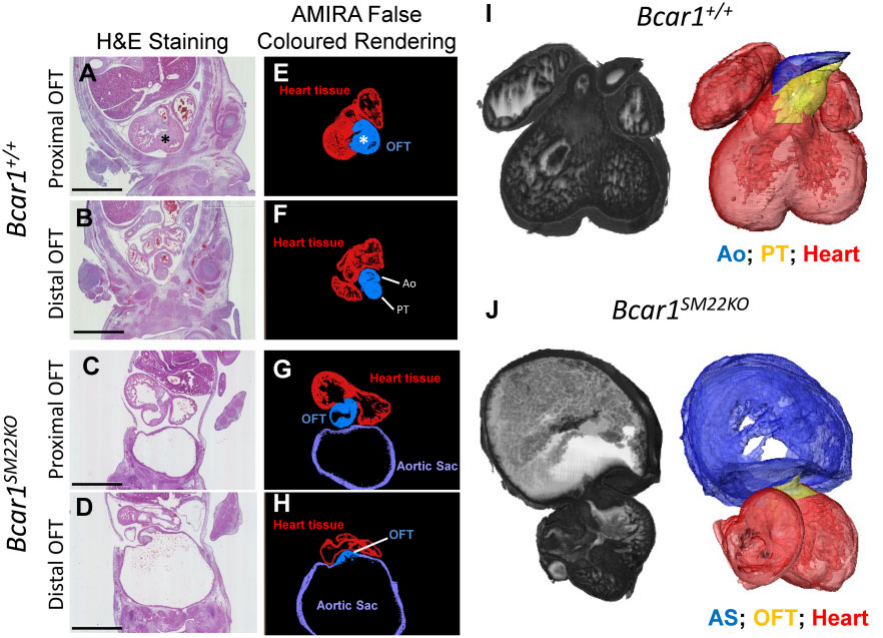
Amira false coloured rendering of OFT septation and HREM volume renderings at E14.5. H&E sections of *Bcar1^+/+^* and *Bcar1^SM22KO^* embryos (*A*–*D*), are presented in false coloured renderings (*E*–*H*), highlighting the OFT (blue), heart tissue (red), and AS (purple); the Ao and pulmonary trunk (PT) are also indicated. (*I* and *J*) 3D grey-scale renderings of E14.5 embryonic hearts, with cut-through to show interior structure, and pseudocoloured surface renderings from HREM data. Ao, aorta; PT, pulmonary trunk. Asterisk in (*A*) and (*E*) highlights the aortico-pulmonary septum. Scale bars are 1 mm.

**Figure 3:**
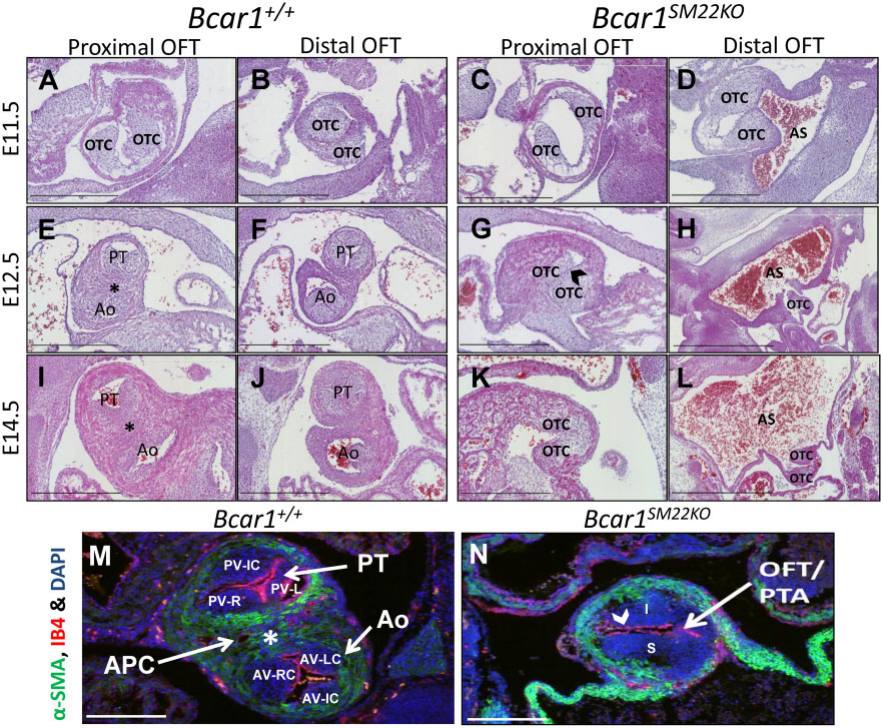
OFT septation defects in *Bcar1^SM22KO^* embryos. (*A*–*D*) OFT septation at E11.5 in littermate controls (*A* and *B*) and *Bcar1^SM22KO^* embryos (*C* and *D*). Proximally, the OTCs in the mutants have migrated less compared to controls; distally the cushions have failed to fuse and the OFT is connected to an abnormally dilated AS. (*E*–*N*) OFT septation at E12.5 (*E*–*H*) and E14.5 (*I*–*N*). Defects in OFT septation in *Bcar1^SM22KO^* embryos were seen throughout the length of the OFT (*G, H, K, L*, and *N*) as compared to littermate controls (*E, F, I, J*, and *M*). In control embryos septation of the OFT is complete and the proximal OTC have fused to form the APC (*E, I*, and *M*). At the site of fusion, a fibrous raphe is seen separating the pulmonary and aortic roots (asterisks in *E* and *I*). This fibrous raphe is transformed into a muscularized septum by E14.5, staining positive for SMA (asterisk in *M*). In *Bcar1^SM22KO^* mutants (*G, K*, and *N*), full separation of the aortic and pulmonary roots and development of the fibrous raphe are not seen, indicated by lack of SMA staining and PTA. Demarcation of the OTC by a layer of endothelial cells is seen in the *Bcar1^SM22KO^* mutants (arrowheads in *G* and *N*). Distally, in the mutants (*H* and *L*), the OFT remains open and connected to an abnormally dilated AS. Whereas in the controls, the Ao and PT are fully separated (*F, J*, and *M*). Immunofluorescent staining of endothelial cells (IB4) and SMA with nuclear DAPI counterstaining is shown in (*M* and *N*). PV‐IC, pulmonary valve intercalated cushion; AV‐IC, aortic valve intercalated cushion; APC, aortico-pulmonary complex; AV‐RC, aortic valve right coronary cusp; AV‐LC, aortic valve left coronary cusp; PV‐R, pulmonary valve right cusp; PV‐L, pulmonary valve left cusp; I, inferior septal cushion; S, superior septal cushion. Scale bars are 400 μm except for (*H* and *L*), which are 900 μm and (*M* and *N*), which are 200 μm.

A failure in OFT septation occurred in *Bcar1^SM22KO^* mutants with 100% penetrance (*Figures 2 and 3* and Supplementary material online, *Figure S6*). These defects were apparent from E11.5 (*Figure 3 A–D*) and became progressively more severe with age. In wild-type E12.5 embryos, the mesenchymal cushions in the proximal OFT had fused and a fibrous raphe [the aortico-pulmonary complex (APC)] had formed between the pulmonary and aortic roots (asterisk in *Figure 3E, I and M*). In contrast, the OTCs in the *Bcar1^SM22KO^* mutants failed to fuse, and remained clearly demarcated by layers of endothelial cells (arrowheads in *Figure 3G and N*), preventing formation of the APC and septum (*Figure 3G, K and N*). In the distal OFT, compared to wild-type embryos (*Figure 3F and J*), separation of the aortic and pulmonary trunks in *Bcar1^SM22KO^* embryos from E12.5 had failed, and the distal OFT was fused with a severely dilated AS (*Figure 3H and L*). By E14.5, septation of the entire OFT had failed in the *Bcar1^SM22K^*^O^ mutants leading to a single outflow vessel resembling persistent truncus arteriosus (PTA),^28^ whereas OFT septation in littermate control embryos was complete (*Figure 2* and Supplementary material online, *Figure S6*).

### 3.4 *Bcar1* deficiency does not impact on PAA development in *Bcar1^SM22KO^* mice but disrupts vascular remodelling

Given the aneurysmal dilatation of the AS in *Bcar1^SM22KO^* mutants and its failure to remodel into the aortic and pulmonary channels, we performed intra-cardiac ink injections to investigate a possible effect on development of the pharyngeal arches and their derivatives, including the aortic arch arteries, which arise from the AS. At E10.5, the third, fourth, and sixth pharyngeal arch arteries were seen in both the *Bcar1^SM22K^*^O^ mutants and their wild-type littermates (Supplementary material online, *Figure S7A–D*), indicating no impairment of PAA development. However, the vessels were significantly dilated in the *Bcar1^SM22KO^* mutants compared to controls (Supplementary material online, *Figure S7E–G*), possibly due to altered blood flow in the mutants. Examination of the vasculature at E13.5 revealed aberrant PAA remodelling evidenced by the presence of the abnormally dilated AS and lack of the carotid and subclavian arteries (Supplementary material online, *Figure S7H and I*).

### 3.5 Effect of *Bcar1* knock out on cell proliferation and migration in the OFT

At E13.5, cell proliferation, detected by immunostaining for the proliferation marker Ki67, was seen throughout the heart and other organs in both wild-type and *Bcar1^SM22KO^* embryos. However, whereas a zone of mainly non-proliferating cells was apparent in wild-type OTCs in the region of the developing septum (asterisk in Supplementary material online, *Figure S8A*), the *Bcar1^SM22KO^* OTCs exhibited strong and generalized Ki67 staining, and lacked a clearly defined area of hypoproliferation (Supplementary material online, *Figure S8B*). Given the known role of myocardialization in OFT septation, we determined whether loss of *Bcar1* could affect cardiomyocyte survival and/or migration into the mesenchymal outlet septum during OTC fusion and septation. We addressed this by immunostaining cardiomyocytes using MF20, which specifically recognizes cardiomyocytes but not VSMC or NCC, and using cleaved caspase-3 as a marker of apoptosis. In wild-type embryos, MF20-positive cardiomyocytes infiltrated deep into the developing septal bridge, whereas *Bcar1^SM22KO^* embryos displayed reduced cardiomyocyte migration into the septum (Supplementary material online, *Figure S8E–H*). No clear change in apoptosis was observed in *Bcar1^SM22KO^* embryos compared with littermate controls (Supplementary material online, *Figure S8C and D*). Quantitation of invasion of septal MF20-positive cells into the OFT revealed a significant decrease in OFT invasion in *Bcar1^SM22KO^* compared with wild-type littermates (Supplementary material online, *Figure S8I*).

We next investigated whether loss of *Bcar1* could affect migration by impacting on expression of key components of the actin cytoskeleton-associated macromolecular machinery that regulates cell migration. Consistent with this possibility, we observed a reduction of F-actin-specific phalloidin staining in cells of the OTCs in the *Bcar1^SM22KO^* mutants compared with littermate controls (Supplementary material online, *Figure S9A and B*), suggesting a disruption in actin cytoskeleton organization in these cells. However, we were unable to detect significantly altered protein expression of Talin, FAK, or Rac1 in protein lysates of OFTs from E11.5 *Bcar1^SM22KO^* embryos compared with wild-type littermate controls (Supplementary material online, *Figure S9C and D*). We also examined expression of Cdc42 (cell division cycle 42), a member of the Rho family of GTPases that acts as a molecular switch to regulate cytoskeleton remodelling and cell movement in cardiovascular development.^29–31^ At E13.5, Cdc42 expression was largely limited to the wild-type OTCs, which had fused and were remodelling to form the muscularized septum separating the aortic and pulmonary channels (arrow in Supplementary material online, *Figure S9E and F*). Strong Cdc42 expression was also present throughout the unfused OTCs of *Bcar1^SM22KO^* embryos (arrows in Supplementary material online, *Figure S9G and H*).

### 3.6 Sm22-restricted *Bcar1* deletion blocks myogenic differentiation and EMT in the OFT

**Figure 4:**
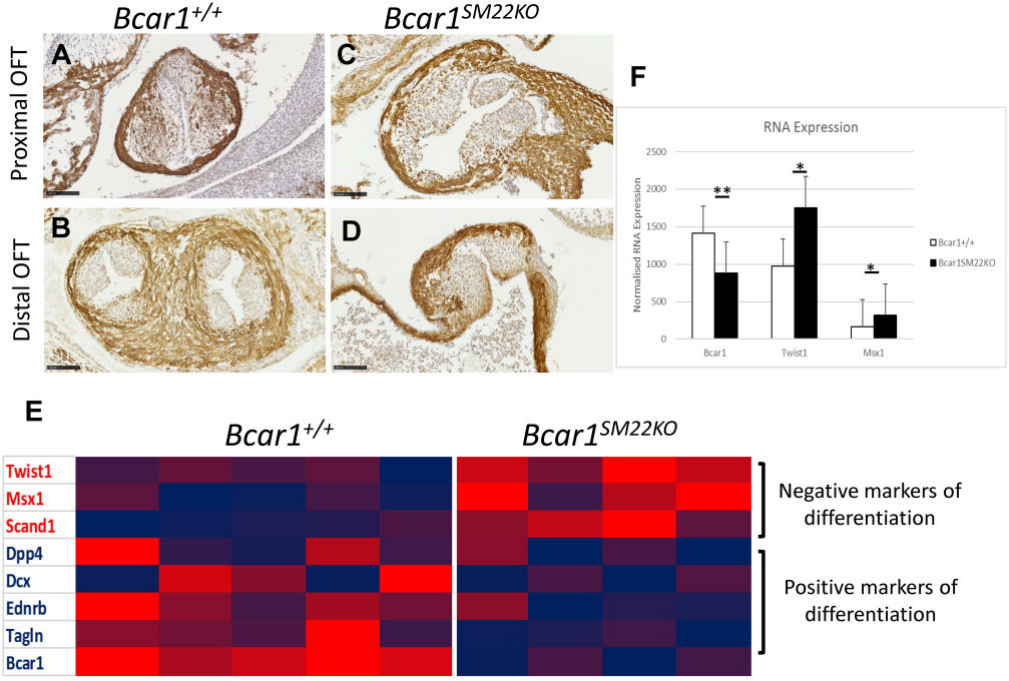
Myogenic differentiation in OTC mesenchyme. Compared to *Bcar1^+/+^* controls (*A*), E12.5 SMA protein expression in the OTC mesenchyme in *Bcar1^SM22KO^* mutants (*C*) was reduced and a muscular septum failed to develop in the distal OFT (*D* vs. *B*). (*E*) Heat-map of differentially regulated genes involved in myogenic differentiation, in RNA lysates from whole OFTs from E11.5 embryos: red represents increased expression, blue decreased expression. (*F*) mRNA expression of *Bcar1, Twist1*, and *Msx1* from OFT RNA lysates from E11.5 *Bcar1^SM22KO^* mutants compared to littermate controls (*Bcar1^+/+^ n*=5, *Bcar1^SM22KO^ n*=4; **P<0.001, *P<0.05, as determined by 2-tailed *t*-test). Scale bars are 100 µm.

The OTCs are populated by mesenchymal cells that migrate from the pharyngeal mesoderm and dorsal neural tube, and subsequently differentiate to smooth muscle actin-expressing cells that form the septum separating the aortic and pulmonary vessels. We hypothesized that the failure in OFT septation in *Bcar1^SM22KO^* mice could arise from an effect of *Bcar1* deficiency on myogenic cell differentiation or EMT. To investigate this possibility, we determined the appearance of mesenchymal cells in the OFT by immunostaining for SMC-specific α-actin (SMA). In wild-type embryos, SMA staining was abundant in the OTCs, localized to the periphery of cells differentiating to form the septum (*Figure 4A and B*), whereas SMA-positive cells were sparse in the *Bcar1^SM22KO^* OTCs and failed to form a septum (*Figure 4C and D*).

To determine the molecular pathways disrupted following *Bcar1* ablation, we performed RNASeq analysis of dissected OFTs from E11.5 *Bcar1^SM22KO^* mutants and littermate controls (Supplementary material online, *Table S4*). This revealed significant up-regulation of several genes encoding transcription factors, including two implicated in inhibition of myogenic differentiation, the basic helix-loop-helix (HLH) transcription factor, *Twist-1*, and the homeobox gene *Msx-1* (Supplementary material online, *Table S4* and *Figure 4E and F*). RNASeq analysis also revealed significant down-regulation of Endothelin Receptor Type B, a signalling pathway which regulates actin cytoskeletal dynamics and is centrally involved in NCC migration in development^32^ (Supplementary material online, *Table S4* and *Figure 4E*). We also observed down-regulation of the mesenchymal differentiation markers *Sm22/Transgelin* and *Myh11* (Supplementary material online, *Table S4* and *Figure 4E*). Gene ontology analysis of RNAseq data revealed an enrichment of genes involved in mesenchymal cell differentiation and cell migration (Supplementary material online, *Table S5*), indicating a role for *Bcar1* in these processes.

**Figure 5:**
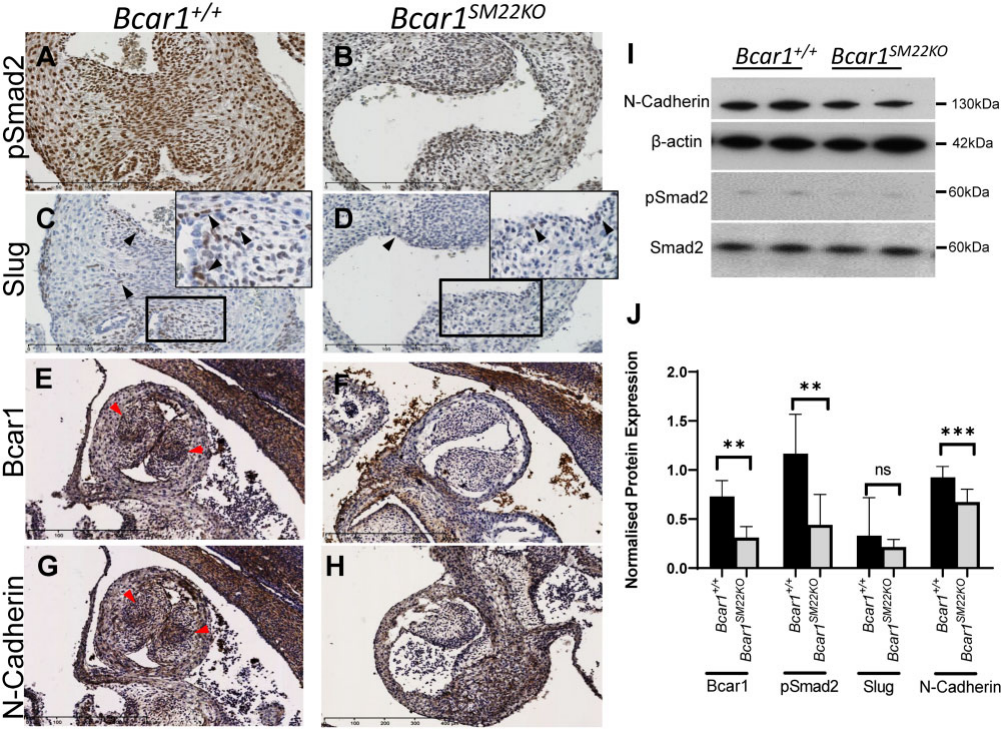
Expression of markers of EMT. Immunostaining of pSmad2 (*A* and *B*), Slug (*C* and *D*), Bcar1 (*E* and *F*), and N-cadherin (*G* and *H*) in OFTs in *Bcar1^+/+^* wild-type and *Bcar1^SM22KO^* littermates (pSmad2 and Slug at E14.5; N-cadherin and Bcar1 at E12.5), magnified views of boxed regions are shown. At E12.5 immunostaining for Bcar1/p130Cas in WT embryos revealed a distinctive whorl-like pattern in the central region of the OTCs (red arrowheads in *E*), which was absent in the *Bcar1^SM22KO^* mutants (*F*). (*I*) Representative western blots of N-cadherin and pSmad2 expression from protein lysates from whole dissected OFTs at E12.5. (*J*) Quantitation of western blot protein bands from E12.5 OFT tissue lysates (**P<0.01, ***P<0.005; *Bcar1^+/+^ n*≥5, *Bcar1^SM22KO^ n*≥5). (*A*–*D*) Scale bars are 200 µm; (*E*–*H*) scale bars are 400 µm.

The effect of *Bcar1* deficiency on EMT was examined by determining the expression of phospho(p)-Smad2, which is a key mediator of TGF-β-induced EMT, and of Slug, an EMT-promoting transcription factor, at E14.5. A marked reduction in pSmad2 expression was seen in the *Bcar1^SM22KO^* embryos compared to the controls, which was particularly apparent in the OTCs (*Figure 5A and B*). Expression of Slug in wild-type embryos was observed in the OTCs (arrowheads in *Figure 5C*), and, concomitant with reduction of pSmad2, was also markedly reduced in the *Bcar1^SM22KO^* mutants (arrowheads in *Figure 5D*).

N-cadherin is expressed in the dorsal region of the neural tube from which CNCCs migrate, and is essential for remodelling of the OFT once CNCCs reach the heart.^33^ Marked N-cadherin expression occurred in the central region of wild-type OTCs very similar to that for *Bcar1*/p130Cas in serial sections (*Figure 5E and G* and Supplementary material online, *Figure S4E*). *Bcar1*/p130Cas and N-cadherin immunostaining was completely absent in *Bcar1^SM22KO^* OFTs (*Figure 5F and H*), indicating loss of *Bcar1* targeted to cells of the developing OFT also expressing N-cadherin, and suggesting a possible role for *Bcar1* in the regulation of N-cadherin expression in CNCCs affecting their ability to differentiate to SMC. Concordant with immunostaining data, western blotting of N-cadherin and pSmad2 expression in OFT lysates from E12.5 embryos revealed a significant reduction in the *Bcar1^SM22KO^* mutants compared to their littermate *Bcar1^+/+^* controls (*Figure 5I and J*). Slug expression was also reduced in the *Bcar1^SM22KO^* mutants but this did not reach statistical significance, possibly due to low Slug expression and the mixed cell population in the tissue lysates analysed.

### 3.7 *Bcar1* deletion in NCC causes defective OFT septation and abnormal AS dilatation

To identify more precisely the role of *Bcar1* in heart development, we generated conditional *Bcar1* knockouts using the *Mef2c-Cre* (*Bcar1^MEF2CKO^*) and *Pax3-Cre* (*Bcar1^PAX3KO^*) transgenic mouse lines, which generate genetic deletions targeted, respectively, to cells that give rise to the second heart field, and to NCC. *Bcar1^MEF2cKO^* mice were born at expected Mendelian ratios, developed to adulthood, and displayed no overt defects (Supplementary material online, *Figure S10A*). In contrast, crossing *Bcar1^fl/fl^* with *Pax3-Cre* mice produced no live homozygous knockout progeny (Supplementary material online, *Figure S10B*).

**Figure 6:**
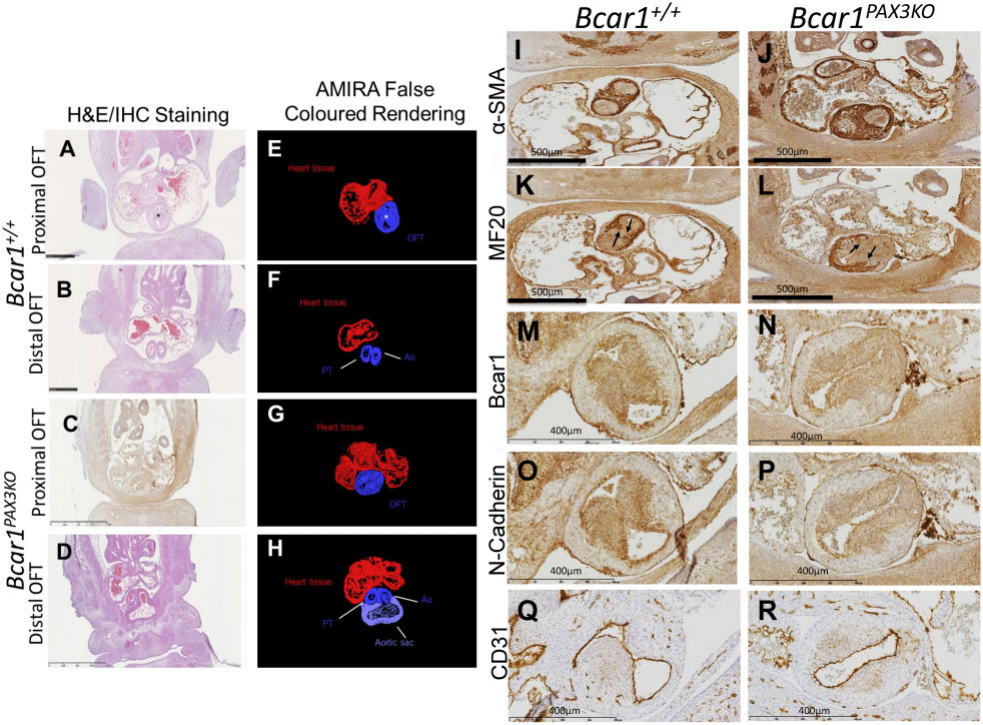
AMIRA false coloured rendering of OFT septation at E12.5 and protein expression in *Bcar1^PAX3KO^* mutant OFTs at E12.5. At E12.5, the APC (asterisk) has formed in the proximal OFT (*A*) and the distal OFT has separated into the Ao and PT (*B*) in the *Bcar1^+/+^* controls, whereas septation of the proximal OFT is incomplete in the *Bcar1^PAX3KO^* mutants (*C*) and distally, the OFT has separated although the Ao remains connected to an abnormally dilated AS (*D*). (*E*–*H*) are false coloured renderings of the tissue sections, highlighting the OFT (blue), heart tissue (red), and the AS (purple). Scale bars in (*A* and *B*) are 500 µm and in (*C* and *D*) are 1 mm. Alpha-SMA expression in the distal OFTs in *Bcar1^PAX3KO^* (*J*) and control littermates (*I*). MF20 immunostaining reveals invasion of cardiomyocytes into the OTC mesenchyme in the *Bcar1^PAX3KO^* mutants (*L*) and littermate controls (*K*). A reduction in Bcar1 (*N*) and N-Cadherin (*P*) expression was seen in the OTC mesenchyme of *Bcar1^PAX3KO^* mutants compared to controls (*M* and *O*). CD31 immunostaining revealed an increase in CD31 +ve cells in the OFTs of the *Bcar1^PAX3KO^* mutants (*R*) compared to littermate controls (*Q*). (*I*–*L*) Scale bars are 500 µm; (*M*–*R*) scale bars are 400 µm.

*Bcar1^PAX3KO^* embryos exhibited aberrant OFT septation, which appeared to be limited to septation of the proximal OFT (Supplementary material online, *Figures S10C–L and S11* and *Figure 6A–H*). An abnormally dilated AS was also observed in *Bcar1^PAX3KO^* mutants. Distally, the OTC mesenchyme in the *Bcar1^PAX3KO^* mutants formed a muscularized septum, indicated by the presence of α-SMA expressing cells (*Figure 6I and J*). Myocardialization of the OFT, examined by MF20 immunostaining, was also apparent in the *Bcar1^PAX3KO^* mutants (arrows in *Figure 6K and L*). Immunostaining of the *Bcar1^PAX3KO^* mutants revealed a reduction in *Bcar1*/p130Cas and N-cadherin expression in the OTC mesenchyme whereas no loss of CD31-positive endothelial cells was detected (*Figure 6M–R*).

## 4. Discussion

This study demonstrates for the first time that targeted deletion of *Bcar1*/p130Cas either in early smooth muscle and cardiac cells, or specifically in NCC, causes severe abnormalities in cardiovascular development, most strikingly in OFT septation and remodelling of the AS. Conditional knock out of *Bcar1/*p130Cas in both SMC and cardiac cells using *SM22-Cre* mice caused an array of developmental cardiovascular anomalies, including a hypoplastic myocardial wall, impaired ventricular development and ventricular septation, a failure of OFT septation resulting in PTA, and abnormal dilatation and remodelling of the AS. The conclusion that these cardiovascular abnormalities are due to direct effects of *Bcar1* deficiency in cardiac cells is supported by the correlation between areas of the heart where the *Bcar1^SM22KO^* mutant embryos show severe defects and with both the regions of prominent *Bcar1* expression in the normal developing heart, namely the ventricles and the OTCs, and the regions of cardiac expression of *SM22* during embryogenesis, such as the presumptive right and left ventricles (LV) and OFT myocardium.^23,^^24^ Whole mouse *Bcar1* knockouts (*Bcar1*-null mutants) displayed a ‘poorly developed heart, consisting of thin myocardium, and prominently dilated blood vessels that retained blood cells’, with prominent expression of *Bcar1* in the wild-type heart and blood vessels from E11.5–12.5.^8^ Our findings of a significantly thinned myocardial wall, dilated AS, and *Bcar1*/p130Cas expression from E11.5 are consistent with the phenotype of *Bcar1*-null mice, though the latter study reported no OFT defects.

*SM22α* transcripts are also expressed in vascular SMC from E9.5 and continue to be expressed in all SMC into adulthood.^22–24^ However, *Bcar1* knock out specifically in SMC using *smMHC-Cre* mice, gave rise to no cardiovascular defects or any overt developmental phenotype and generated viable homozygous mice. It is noteworthy that conditional deletion of *Bcar1*/p130Cas in skeletal muscle using the muscle creatine kinase (*Ckmm*)*-Cre* transgenic line also produced phenotypically normal, viable, and fertile mice in normal Mendelian ratios.^10^ Targeted ablation of *Bcar1* in endothelial cells and second heart field cells also had no developmental or post-natal effects. In contrast, NCC-specific *Bcar1* deletion in *Bcar1^PAX3KO^* mice partially recapitulated the cardiac defects in *Bcar1^SM22KO^* mutants, including proximal OFT septation defects and abnormal dilatation of the AS. The overlap in defects seen in the *Bcar1^SM22KO^* and *Bcar1^PAX3KO^* mutants highlight a role for *Bcar1* specifically in NCC relevant for OFT septation and septum formation. The results also indicate that other cell types, including epicardial, endocardial, and other cardiomyocyte progenitor cells, may be responsible for the ventricular remodelling and proximal OFT septation defects observed in the *Bcar1^SM22KO^* mutants but not the *Bcar1^PAX3KO^* mutants. Identifying these other specific cell types will be the focus of future experimental work. In contrast, *Bcar1* is not required for SMC or endothelial cell development or maturation, in second heart field cells, or for adult SMC function in the Ao or other heart vessels.

The conclusion that *Bcar1* plays a key role in OFT septation and ventricular development is supported by the spatiotemporal correlation between these processes and dynamic changes in Bcar1/p130Cas protein expression in the embryonic heart. In wild-type embryos, Bcar1/p130Cas expression was first observed in the OTCs at E11.5, coincident with the onset of distal OFT septation, formation of the spiralling OTCs, and NCC insertion into the AS to divide the aortic and pulmonary channels.^26^ Bcar1/p130Cas expression in OTCs was prominent at E12.5, when septation of the proximal OFT progresses, but had markedly declined by E13.5 when OFT septation is complete, consistent with a dynamic and time-specific role for *Bcar1* in the cardiac cell contributions to OFT septation. A similar spatiotemporal correlation is evident between ventricular Bcar1/p130Cas expression and ventricular septation, myocardial compaction, trabeculation and increased ventricular wall thickness, phenotypic characteristics impaired in *Bcar1^SM22KO^* mice, and all of which occur between E11.5 and E13.5. Given that NCC-specific N-cadherin deficiency generated using *Wnt1-Cre* mice also causes a lethal failure of normal OFT septation,^33^ the loss of N-Cadherin expression caused by *Bcar1* deletion in *Bcar1^SM22KO^* and *Bcar1^PAX3KO^* mice is a possible mechanism underlying the failure of OFT septation. This is supported by the marked similarity in the immunostaining for N-cadherin and *Bcar1*/p130Cas in the OTCs, which strongly resemble the infiltrating columns of CNCCs during OFT septation, and the OFT septation defects seen in the NCC-specific *Bcar1^PAX3KO^* mutants. These findings suggest that *SM22-Cre* and *Pax3-Cre* may drive conditional *Bcar1* deletion in overlapping NCC populations, though confirmation of this will require further experimental work.

The embryonic lethality of *Bcar1^SM22KO^* and *Bcar1^PAX3KO^* mice from E14.5 to 15.5 probably arises from the failure of OFT septation and abnormal AS dilatation leading to inadequate blood supply to the developing embryo. Another notable feature of *Bcar1^SM22KO^* mutants likely contributing to their embryonic lethality was defective compaction of the ventricular myocardium, associated with impaired development of the network of trabeculations in the ‘spongy layer’ during compaction, which increases myocardial oxygenation in the absence of the coronary vessels.^34^ Compaction of the ventricular myocardium, occurring between E13 and E14 in the mouse, is important for function of the ventricles during the later foetal stages, and its disruption is linked to heart failure and sudden cardiac death.^26^

Differentiation of myogenic and mesenchymal cells and endocardial EMT play essential roles in heart development, particularly in the heart valves and the OTCs.^35^ Septation of the proximal OFT requires both the transformation of endocardial cells into mesenchymal cells (endocardial EMT) that populate the cardiac jelly, and invasion of the OTCs by neural crest cells.^14,^^26,^^36,^^37^ This study presents several lines of evidence that *Bcar1* plays a key role in mesenchymal differentiation and EMT in OFT septation. The absence of SMA-positive OTC cells in *Bcar1^SM22KO^* mutants demonstrates a marked impairment in mesenchymal differentiation. The increase in proliferation indicated by increased Ki67 staining in *Bcar1^SM22K^*^O^ OTC cells and the lack of a clear septal zone of hypoproliferation, suggests that loss of *Bcar1* resulted in deregulation of cell proliferation possibly arising from a failure to arrest proliferation precedent to differentiation. Disrupted EMT is also supported by the marked reduction in Smad2 phosphorylation and in Slug expression in the OTCs observed in mutants compared with wild-type embryos. TGF-β, an essential inducer of EMT in cardiac development, induces SMA expression in NCC, while Smad2 phosphorylation is a key mediator in TGF-β signalling.^38,^^39^ Slug transcription factor also plays a central role in EMT and in neural crest emigration.^40^ For example, silencing of Slug expression in chick embryos causes mesodermal malformation and neural crest emigration failure.^40^ Reduced SMA expression, and loss of Smad2 phosphorylation and downstream Slug expression are therefore likely to result in suppression of EMT. RNASeq analysis of *Bcar1^SM22KO^* OFTs at E11.5 showed that ablation of *Bcar1* resulted in up-regulation of *Twist1* and *Msx1*, transcription factors implicated in negative regulation of myogenic cell differentiation. During myogenesis, down-regulation of *Twist1* in developing somites occurs co-ordinately with up-regulation of the myogenic transcription factors MyoD and Myf5, which play key roles in the later stages of muscle development,^41^ and Twist1 inhibited muscle cell differentiation in mouse C2C12 mouse myoblasts and in embryoid bodies derived from human embryonic stem cells.^42,^^43^ During development, Msx1 expression is associated with regions of highly proliferative and multipotent cells, and inversely correlated with cell differentiation and exit from the cell cycle, in limb buds, e.g.^44^ Furthermore, *Msx1* overexpression inhibits myogenic differentiation in cell culture models and *in vivo*, and represses expression of MyoD and other myogenic transcription factors.^45,^^46^ Targeted *Bcar1* deletion also resulted in down-regulation of Endothelin Receptor Type B, which has a role in embryonic NCC migration and in signalling pathways that regulate actin cytoskeletal organization,^32^ as well as markers of smooth muscle differentiation, such as Sm22/transgelin. These changes in gene expression taken together with the severe impairment of mesenchymal cell maturation and decreased expression of markers of EMT in *Bcar1^SM22KO^*, suggest that during normal heart development, *Bcar1* plays a key role in maintaining a microenvironment permissive of myogenic and mesenchymal differentiation, in part by negatively regulating transcriptional networks involving *Twist1* and *Msx1*, and may also play a role in NCC migration through the positive regulation of pro-migratory pathways. Though it is unclear how *Bcar1/*p130Cas regulates OFT gene expression, it is noteworthy that the C-terminal *Bcar1/*p130Cas region contains a HLH domain,^1,^^47^ and a 31 kDa *Bcar1/*p130Cas C-terminal cleavage product containing the HLH domain heterodimerized with the basic HLH transcription factor, E2A, translocated to the nucleus and inhibited E2A-mediated p21(Waf1/Cip1) transcription.^48,^^49^ Further work is warranted to elucidate whether *Bcar1/*p130Cas and/or its cleavage products could similarly mediate direct or indirect effects on gene expression programmes during cardiac OFT remodelling.

A dilated AS was a striking feature of *Bcar1^SM22KO^* and *Bcar1^PAX3KO^* mice that may result from an impact on SMC progenitors, particularly NCC-derived SMC. For example, *SM22*-restricted ablation of Integrin-linked kinase caused aortic aneurysm associated with perinatal lethality between E18.5 and P1, with no defects in OFT septation or ventricular development.^50^ This suggests that *SM22*-specific knock out can affect AS remodelling independently of effects on the progenitor cell populations contributing to OFT and ventricular remodelling, most likely through an impact on SMC and their progenitors. The lack of phenotype in SMC-specific *Bcar1^SMKO^* mice indicates that the aortic aneurysm in *Bcar1^SM22KO^* mice may not be due to SMC-specific loss, a conclusion supported by our observation that loss of *Bcar1* did not prevent migration or subsequent differentiation of SMC progenitors essential for formation of the pharyngeal arch arteries. However, the dilated AS in *Bcar1^SM22KO^* mice may reflect impaired function of a subpopulation of SMC progenitors unaffected by *smMHC-*restricted ablation and distinct from those responsible for remodelling of pharyngeal arch arteries. AS dilatation may also be secondary to haemodynamic changes arising from earlier aberrant ventricular and/or OFT development due to *Bcar1* deficiency in NCC and other cardiac cells.

The finding that conditional deletion of *Bcar1* impairs heart development in mice has implications for the aetiology of human heart defects. A marked feature of *Bcar1^SM22KO^* mice was a failure of fusion of the OTCs and of the development of the fibrous raphe, resulting in formation of a single outflow vessel. This is a salient feature of PTA, which is a hallmark of several human congenital heart diseases, including DiGeorge syndrome (also known as del22q11).^28^ DiGeorge syndrome results from heterozygous deletions within human chromosome 22q11, and it is of interest for this study that one of the genes most strongly involved in these deletions is *v-crk* sarcoma virus CT10 oncogene homolog (avian)-like (CrkL), closely related to Crk, which is known to associate with p130Cas (Cas denotes Crk-associated substrate). *Bcar1* is located on human chromosome 16, and not predicted to be perturbed by heterozygous deletions within 22q11. However, many congenital heart defects are of unknown aetiology, and may result from interactions between rare and largely unknown genetic defects. Tetralogy of Fallot (TOF) is the most common human cyanotic heart malformation, but only 20% of TOF patients have defined genetic causes, such as DiGeorge syndrome. A recent study of single-gene variants in a well-characterized TOF patient cohort, identified several novel null variants including a deletion of exons 2–7 in *Bcar1*, and a stopgain variant of *IQGAP1*,^51^ a gene encoding for an adapter protein that associates with Bcar1/p130Cas in endothelial cells.^52^ Another study reported a loss-of-function variant in *Bcar1* in patients with conotruncal defects.^53^ These findings indicate that functional loss of *Bcar1* and associated signalling networks plays a role in the aetiology of some human congenital heart defects, and suggest that mice with conditional genetic disruption of *Bcar1* will prove useful for dissecting underlying mechanisms involved in the causation of such congenital heart abnormalities.

## Data availability

The data underlying this article are available in the article and in its Supplementary material online.

## Supplementary material

Supplementary material is available at *Cardiovascular Research* online.

## Supplementary Material

cvab242_Supplementary_Data

## Notes

Translational perspective The molecular pathways coordinating cardiogenesis and the remodelling of the OFT are complex, and dysregulation of these pathways causes human heart defects. Our findings highlight a specific requirement for *Bcar1* essential for cardiogenesis. Furthermore, the failure of OFT septation in *Bcar1^SM22KO^* mice resembles PTA, a feature of several human congenital heart diseases, including DiGeorge syndrome. Our findings have implications for the mechanisms underlying the pathogenesis of congenital heart disease, and suggest that mice with conditional *Bcar1* deletions may be useful models for dissecting mechanisms involved in the pathogenesis of human heart defects.
