# Supplementary material for: Bcar1/p130Cas is essential for ventricular development and neural crest cell remodelling of the cardiac outflow tract
Source: Cardiovasc Res. 2021 Jul 16;118(8):1993–2005. doi: 10.1093/cvr/cvab242 (PMC9239580; doi:10.1093/cvr/cvab242)
Supplement: cvab242_Supplementary_Data [file cvab242_supplementary_data.zip › Mahmoud et al. Supplementary (updated fig.9).pdf]

Bcar1/p130Cas is essential for ventricular development and neural crest cell remodelling of the cardiac outflow tract

Short title: *Bcar1* in ventricular and outflow tract remodelling.

Marwa Mahmoud [1], Ian Evans [1#], Laura Wisniewski [1$], Yuen Tam [1], Claire Walsh [2], Simon Walker-Samuel [2], Paul Frankel [3], Peter Scambler [4], and Ian Zachary [1]

[1]Centre for Cardiometabolic and Vascular Science, BHF Laboratories, UCL Division of Medicine, 5 University Street, London WC1E 6JF, United Kingdom.

[2] UCL Centre for Advanced Biomedical Imaging, Paul O'Gorman Building, 72 Huntley Street, London WC1E 6DD, United Kingdom.

[3] Institute of Cardiovascular Science, University College London, 5 University Street, London WC1E 6JF, United Kingdom.

[4] Developmental Biology of Birth Defects Section, UCL Institute of Child Health, 30 Guilford Street, London WC1N 1EH, United Kingdom.

[#]Current address: The Francis Crick Institute, London NW1 1AT, UK.

[$]Current address: Queen Mary University of London, London EC1M 6BQ, UK.

Corresponding Authors:

Ian Zachary, BHF Laboratories, UCL Division of Medicine, Rayne Building, 5 University St, London WC1E 6JF, UK.

E-mail: i.zachary@ucl.ac.uk

Marwa Mahmoud, BHF Laboratories, UCL Division of Medicine, Rayne Building, 5 University St, London WC1E 6JF, UK.

E-mail: marwa.mahmoud@ucl.ac.uk

Supplementary Data

Supplementary Table 1. Details of genotyping primers.

Genotyping Primers	Gene Targeted	Sequence (5'-3')
FlP130Cas_F1<br>FlP130Cas_R1	floxed *Bcar1/ p130Cas* allele	5'- GTTGCGAGGACCCTAATACCTCAATCC-3'<br>5'- TGGCTCCCGTACCTGAAATCAAACC-3'
rP130Cas_F2<br>rP130Cas_R2	recombined *Bcar1/p130Cas* allele	5'- ACTGATAAAGCCAGTAGCATCCAGTCACG-3'<br>5'- CATTCTGCTCTCCAGCAGCTATGGG-3'
Sm22Cre_F<br>Sm22Cre_R	*Sm22-Cre transgene*	5'- GCTGCCACGACCAAGTGACAGCAATG-3'<br>5'- GTAGTTATTCGGATCATCAGCTACAC-3'
Smmhc-Cre_F<br>Smmhc-Cre_R	*Smmhc-Cre* transgene	5'-CCA ATT TAC TGA CCG TAC ACC-3'<br>5'-GTT TCA CTA TCC AGG TTA CGG-3'
Tie2-Cre_F<br>Tie2-Cre_R	*Tie2-Cre* transgene	5'-CGCATAACCAGTGAAACAGCATTGC-3'<br>5'-CCCTGTGCTCAGACAGAAATGAGA-3'
LacZ1<br>LacZ2<br>LacZ3	*LacZ* allele	5'-AAA GTC GCT CTG AGT TGT TAT-3'<br>5'-GGA GCG GGA GAA ATG GAT ATG-3'<br>5'-GCG AAG AGT TTG TCC TCA ACC-3'
Mef2c-Cre F<br>Mef2c-Cre R	*Mef2c-Cre* transgene	5´-TGC CAC GAC CAA GTG ACA GC-3´<br>5´-CCA GGT TAC GGA TAT AGT TCA TG-3´
Pax3-Cre 1<br>Pax3-Cre 2<br>Pax3-Cre 3	*Pax3-Cre* transgene	5'-AGG CAA ATT TTG GTG TAC GG-3'<br>5'-CTG CAC TCA AGG GAC TCC TC-3'<br>5'-GTG AAG GCG AGA CGA AAA AG-3'

Supplementary Table 2. Antibodies used for Western blotting.

Protein	Antibody Details	Working Dilution
Gapdh	Goat polyclonal, Santa Cruz # sc-20357	1/2,500
Beta-actin	Mouse monoclonal, Sigma #A5441	1/2,500
Talin	Rabbit monoclonal, Cell Signalling #4021	1/250
Bcar1/p130Cas	Mouse monoclonal, BD Biosciences #610272	1/500
FAK	Rabbit monoclonal, Cell Signalling #3285	1/250
Rac1	Mouse monoclonal, Millipore #05-389	1/1,000
pSmad2	Rabbit monoclonal, Cell Signalling #3108.	1/250
N-Cadherin	Mouse monoclonal, Santa Cruz #sc- 59987	1/500
Slug	Rabbit monoclonal, Cell Signalling #9585.	1/250

Supplementary Table 3. Antibodies used for immunohistology.

Protein	Antibody Details	Working Dilution	Antigen Retrieval/ Special Kit Used
α-SMA	Monoclonal Anti-Actin, α-Smooth Muscle, clone 1A4. Sigma #A2547.	1/100	M.O.M Kit (Vector Labs #BMK-2202)
Bcar1/p130Cas	Mouse monoclonal, BD Biosciences #610272.	1/50	M.O.M Kit (Vector Labs #BMK-2202)
MF20	Mouse monoclonal, Novus Biologicals # MAB4470.	1/100	M.O.M Kit (Vector Labs #BMK-2202)
pSmad2	Rabbit monoclonal, Cell Signalling #3108.	1/100	Citrate buffer (pH6.0)
Slug	Rabbit monoclonal, Cell Signalling #9585.	1/100	Citrate buffer (pH6.0)
Cdc42	Rabbit monoclonal, Cell Signalling # #2466.	1/100	Citrate buffer (pH6.0)
Phalloidin-Alexa 594	Alexa Fluor™ 594 Phalloidin, Thermo Fisher # A12381.	1/400	
CD31	Purified Rat Anti-Mouse CD31 Clone MEC 13.3, BD Biosciences # 550274.	1/100	
Isolectin B4_Dylight 594	DyLight 594 Labeled Griffonia Simplicifolia Lectin I (GSL I)	1/100	

	isolectin B4. Vector Labs #DL-1207.		
α-SMA_FITC	Monoclonal Anti-Actin, α-Smooth Muscle - FITC antibody, clone 1A4. Sigma #F3777.	1/100	
N-Cadherin	Mouse monoclonal, Santa Cruz #sc-8424.	1/50	M.O.M Kit (Vector Labs #BMK-2202)
Ki67	Ki-67 (D3B5) Rabbit pAb. Cell Signaling Technology #12202.	1/200	Citrate buffer (pH6.0)
Cleaved Caspase-3	Cleaved Caspase-3 (Asp175) Rabbit pAb. Cell Signaling Technology #9661.	1/200	Citrate buffer (pH6.0)

Supplementary Table 4. RNASeq analysis of *Bcar1*^{SM22KO} OFT RNA lysates compared to *Bcar1*$^{+/+}$ littermate controls. Genes with a raw p-value <0.01 and a log2 fold change >0.5 or <-0.5 are listed.

**Gene Id**	***Bcar1*** $^{+/+}$ **Group Mean**	***Bcar1*** ^{SM22KO} **Group Mean**	**Fold Change**	**log2Fold Change**	**Raw p-value**	**Adj. p-value**
Npb	15	34	2.2667	1.1806	0.00979	0.6388
Sep-03	706	486	0.6884	-0.5387	0.00979	0.6388
Dpt	1	13	13.0000	3.7004	0.00971	0.6388
Metrnl	56	82	1.4643	0.5502	0.00918	0.6161
Lgals7	10	24	2.4000	1.2630	0.00917	0.6161
C1qtnf4	41	70	1.7073	0.7717	0.00916	0.6161
Shroom1	132	198	1.5000	0.5850	0.00904	0.6161
Bnc1	302	167	0.5530	-0.8547	0.00893	0.6161
Gbp7	45	25	0.5556	-0.8480	0.00880	0.6161
Col2a1	6649	9510	1.4303	0.5163	0.00849	0.6098
Lama1	305	202	0.6623	-0.5945	0.00846	0.6098
Fam101a	46	84	1.8261	0.8688	0.00837	0.6098
Nmnat2	53	34	0.6415	-0.6405	0.00835	0.6098
A730046J19Rik	9	3	0.3333	-1.5850	0.00814	0.6098
Trpm2	2	7	3.5000	1.8074	0.00779	NA
Zhx3	171	120	0.7018	-0.5110	0.00707	0.5799

Tshz3	255	166	0.6510	-0.6193	0.00697	0.5754
4930596I21Rik	1	6	6.0000	2.5850	0.00680	NA
Gp1bb	32	50	1.5625	0.6439	0.00680	0.5698
Hmgcs2	309	459	1.4854	0.5709	0.00676	0.5698
Gm1673	63	100	1.5873	0.6666	0.00638	0.5550
Tcf15	34	57	1.6765	0.7454	0.00614	0.5550
Fdx1l	16	29	1.8125	0.8580	0.00612	0.5550
Lcor	42	24	0.5714	-0.8074	0.00606	0.5550
Skap2	333	208	0.6246	-0.6789	0.00603	0.5550
Pcdh12	213	150	0.7042	-0.5059	0.00602	0.5550
Col9a2	712	1270	1.7837	0.8349	0.00593	0.5550
Myocd	698	460	0.6590	-0.6016	0.00577	0.5550
Ngfr	442	303	0.6855	-0.5447	0.00560	0.5540
Prr19	6	12	2.0000	1.0000	0.00541	0.5431
Col19a1	8	16	2.0000	1.0000	0.00523	0.5322
1700001L05Rik	91	62	0.6813	-0.5536	0.00515	0.5293
Tlx2	15	3	0.2000	-2.3219	0.00511	0.5293
Lrrn4	451	315	0.6984	-0.5178	0.00511	0.5293
Bmp6	164	236	1.4390	0.5251	0.00454	0.5027
Scx	165	304	1.8424	0.8816	0.00440	0.4979
Hmga1	15	29	1.9333	0.9511	0.00435	0.4970
Myh11	135	77	0.5704	-0.8100	0.00400	0.4669
Gbp8	15	6	0.4000	-1.3219	0.00398	0.4669

Efhb	10	3	0.3000	-1.7370	0.00397	0.4669
Sema3d	255	140	0.5490	-0.8651	0.00394	0.4669
Scn2b	170	120	0.7059	-0.5025	0.00382	0.4669
1500017E21Rik	52	35	0.6731	-0.5712	0.00378	0.4669
Gjb6	3	14	4.6667	2.2224	0.00361	0.4538
Ppp1r35	179	266	1.4860	0.5715	0.00351	0.4487
Rgs9	92	62	0.6739	-0.5694	0.00348	0.4487
Scel	23	9	0.3913	-1.3536	0.00334	0.4414
Shtn1	76	38	0.5000	-1.0000	0.00313	0.4294
Miat	98	36	0.3673	-1.4448	0.00308	0.4257
Spint2	503	716	1.4235	0.5094	0.00306	0.4257
Osr1	648	298	0.4599	-1.1207	0.00300	0.4257
Atp10b	13	3	0.2308	-2.1155	0.00299	0.4257
Mctp2	17	6	0.3529	-1.5025	0.00295	0.4257
Maf	223	128	0.5740	-0.8009	0.00286	0.4239
Zeb2	760	506	0.6658	-0.5869	0.00269	0.4061
Lbx1	1	7	7.0000	2.8074	0.00248	NA
C130021I20Rik	1	7	7.0000	2.8074	0.00245	NA
Gabrp	4	21	5.2500	2.3923	0.00236	0.3711
Eva1b	320	465	1.4531	0.5392	0.00228	0.3659
Prr7	29	48	1.6552	0.7270	0.00219	0.3577
Adam33	67	42	0.6269	-0.6738	0.00208	0.3460
Edn1	260	394	1.5154	0.5997	0.00200	0.3441

Slitrk6	77	31	0.4026	-1.3126	0.00196	0.3441
Sema3g	130	86	0.6615	-0.5961	0.00191	0.3441
Serpinb9	214	143	0.6682	-0.5816	0.00186	0.3428
Adcy8	41	18	0.4390	-1.1876	0.00184	0.3428
Etv4	403	674	1.6725	0.7420	0.00173	0.3428
Col9a3	836	1336	1.5981	0.6763	0.00166	0.3428
Tpgs1	196	279	1.4235	0.5094	0.00165	0.3428
Zfp771	117	185	1.5812	0.6610	0.00165	0.3428
Jund	2168	3126	1.4419	0.5280	0.00162	0.3428
Bri3	473	676	1.4292	0.5152	0.00162	0.3428
Cemip	178	118	0.6629	-0.5931	0.00162	0.3428
Ret	105	28	0.2667	-1.9069	0.00151	0.3428
Thbd	514	323	0.6284	-0.6702	0.00148	0.3428
Prss37	6	19	3.1667	1.6630	0.00147	0.3428
Disp2	17	4	0.2353	-2.0875	0.00146	0.3428
Rasgrp3	368	258	0.7011	-0.5123	0.00143	0.3428
Jdp2	130	184	1.4154	0.5012	0.00125	0.3428
Ptprt	49	26	0.5306	-0.9143	0.00123	0.3428
Kcne1	505	749	1.4832	0.5687	0.00123	0.3428
Msx2	203	296	1.4581	0.5441	0.00115	0.3428
Etohi1	51	27	0.5294	-0.9175	0.00108	0.3428
Chga	69	3	0.0435	-4.5236	0.00099	0.3365
Sema3e	46	16	0.3478	-1.5236	0.00099	0.3365
Clic5	172	121	0.7035	-0.5074	0.00093	0.3298

Ier5l	155	227	1.4645	0.5504	0.00090	0.3264
Crlf2	69	108	1.5652	0.6464	0.00084	0.3264
Wnt6	10	44	4.4000	2.1375	0.00071	0.2942
Sc5d	458	322	0.7031	-0.5083	0.00057	0.2504
Tshz1	961	634	0.6597	-0.6001	0.00056	0.2504
Afap1l2	102	46	0.4510	-1.1489	0.00049	0.2409
Pcolce	1859	2653	1.4271	0.5131	0.00048	0.2409
Mylk	652	363	0.5567	-0.8449	0.00048	0.2409
Dbh	29	4	0.1379	-2.8580	0.00043	0.2409
Rnaset2b	15	36	2.4000	1.2630	0.00034	0.2218
Ccdc88c	190	126	0.6632	-0.5926	0.00021	0.1588
Id1	1112	1726	1.5522	0.6343	0.00020	0.1588
Pdzd2	334	226	0.6766	-0.5635	0.00016	0.1442
Gpr50	4	14	3.5000	1.8074	0.00015	0.1424
Gucy1a3	1002	548	0.5469	-0.8706	0.00014	0.1414
Phox2b	86	16	0.1860	-2.4263	0.00011	0.1207
**Dpp4**	**220**	**145**	**0.6591**	**-0.6015**	**0.00004**	**0.0490**
**Twist1**	**972**	**1750**	**1.8004**	**0.8483**	**0.00004**	**0.0490**
**Gbp4**	**60**	**31**	**0.5167**	**-0.9527**	**0.00003**	**0.0444**
**Tecrl**	**185**	**126**	**0.6811**	**-0.5541**	**0.00003**	**0.0431**
**Dcx**	**149**	**49**	**0.3289**	**-1.6045**	**0.00002**	**0.0324**
**Msx1**	**167**	**321**	**1.9222**	**0.9427**	**0.00001**	**0.0274**
**Ednrb**	**977**	**500**	**0.5118**	**-0.9664**	**0.00001**	**0.0257**
**Grik3**	**100**	**50**	**0.5000**	**-1.0000**	**0.00000**	**0.0104**

**Tagln**	**8627**	**4671**	**0.5414**	**-0.8851**	**0.00000**	**0.0071**
**Scand1**	**154**	**326**	**2.1169**	**1.0819**	**0.00000**	**0.0071**
**Bcar1**	**1410**	**886**	**0.6284**	**-0.6703**	**0.00000**	**0.0000<br>1**

Supplementary Table 5. Gene Ontology term enrichment analysis. Table showing GO terms enriched >10 fold in the RNASeq gene list.

Analysis Summary:

Analysis Type: PANTHER Overrepresentation Test (Released 20190711)

Annotation Version and Release Date: GO Ontology database Released 2019-12-09

Reference List: Mus musculus (all genes in database)

Annotation Data Set: GO biological process complete

Test Type: Fisher's Exact

Correction: Bonferroni-correction

Displaying only results for Bonferroni-corrected for P < 0.05

GO biological process complete	Mus musculus (REF) #	Gene list #	expected	Fold Enrichment	P value
enteric nervous system development	14	4	0.07	+59.45	1.28E-02
neural crest cell migration	55	8	0.26	+30.27	5.80E-06
ameboidal-type cell migration	174	9	0.84	+10.76	2.15E-03

neural crest cell development	80	9	0.38	+23.41	3.82E-06
stem cell development	86	9	0.41	+21.78	6.89E-06
stem cell differentiation	166	14	0.8	+17.55	1.61E-09
neural crest cell differentiation	87	9	0.42	+21.53	7.58E-06
mesenchymal cell differentiation	150	12	0.72	+16.65	1.67E-07
mesenchyme development	210	14	1.01	+13.87	3.21E-08
mesenchymal cell development	85	9	0.41	+22.03	6.26E-06
cellular response to BMP stimulus	93	7	0.45	+15.66	4.63E-03

Supplementary Figure Legends

Supplementary Figure 1. Generation of the conditional *Bcar1* floxed mouse.

A) Targeting strategy. Schematic representation of the wild type, recombined and Flp-mediated excised *Bcar1* alleles with the relevant restriction sites for the Southern blot analysis and the primer locations for the PCR analysis.

B) Southern blot analysis. Genomic DNA of the recombined ES cell clones was compared with wild-type DNA (C57BL/6). The digested DNAs were blotted on nylon membrane and hybridised with the 5' probe detecting the BamHI-fragment to screen for the 5' homologous recombination event.

C) Targeting strategy used to generate the *Bcar1* conditional knockouts.

Supplementary Figure 2. Characterisation of *Bcar1* conditional knockout mice.

(A) X-Gal staining showing *SM22-Cre* activity in the right ventricle (RV), left ventricle (LV), and outflow tract (OFT) at E9. Boxed region is magnified view showing *Cre* activity in the epicardium (arrow, scale bar is 50μm).

(B) Genotyping of embryos. PCR detects the *Bcar1* WT allele (385bp) or/and the $Bcar1^{fl}$, floxed, allele (478bp) (top gel). A 400bp band indicates the presence of the *SM22α-Cre* transgene (middle gel). Cre-mediated recombination of floxed *Bcar1* generates a 697bp product corresponding to the *Bcar1* recombined allele (bottom gel).

(C) Western blot analysis. Reduced Bcar1 protein levels in lysates from the right ventricle and OFT tissue from E11.5 $Bcar1^{SM22KO}$ embryos compared to littermate $Bcar1^{+/+}$ controls.

(D) X-Gal staining of heart tissue from postnatal day 7 mice showing specific *smMHC-Cre* expression in vascular smooth muscle cells (arrowheads) surrounding the vasculature.

(E) Genotyping of adult mice. A band of 900bp indicates the presence of the *smMHC-Cre* transgene (top gel). PCR to detect the *Bcar1* allele generates a 385bp (*Bcar1* WT) or/and a 478bp (*Bcar1*^{fl}, floxed) product (middle gel). Cre-mediated recombination of floxed *Bcar1* generates a 697bp product corresponding to the *Bcar1* recombined allele (bottom gel).

(F) *Bcar1*/p130Cas western blots of protein lysates from the aortas of adult (≥ 4 week-old) *Bcar1*^{SMKO} and littermate *Bcar1*$^{+/+}$ control mice.

Supplementary Figure 3. Mendelian ratios and gross phenotypes of *Bcar1* conditional knockouts.

(A) *SM22-Cre*–restricted knockout of *Bcar1* results in embryonic lethality.

(B) E14.5 *Bcar1*^{SM22KO} embryos showed signs of severe morbidity including evidence of an abnormally developed heart compared to littermate *Bcar1*$^{+/+}$ controls (arrowheads), and haemorrhaging (hollow arrowhead). Scale bars are 2mm.

(C) Mendelian ratios. Endothelial cell – specific knockout of *Bcar1* results in viable *Bcar1*^{TIE2KO} mice that are born at the expected Mendelian ratio. Smooth muscle cell – specific knockout of *Bcar1* results in viable *Bcar1*^{SMKO} mice born at the expected Mendelian ratio. Actual numbers of mice are shown in brackets.

(D) Histological analysis of the vasculature in heart and lung tissue from postnatal day 9 mice revealed no gross morphological defects in the vessels (arrowheads).

Supplementary Figure 4. Bcar1 protein expression in control *Bcar1$^{+/+}$* embryos.

At E10.5 no Bcar1 protein expression was detected by immunohistochemical staining in the heart (A,B). By E11.5 Bcar1 is detected in the OFT cushion mesenchyme (C,D) and in the endocardium (I).This expression dramatically increases at E12.5 (arrowheads in E,F), strong expression in the epicardium and endocardium, and some weak expression in the trabecular myocardium is also detected at E12.5 (J). By E13.5 expression of Bcar1 in the outflow tract septum has decreased and is limited to the epicardium, ventricular myocardium and the mesenchyme of the remodelling aortic and pulmonary valve leaflets (G,H). (A,C,E,F,G,H) scale bars are 250µm; (B,D) scale bars are 100µm, (I,J) scale bars are 50µm. All images are representative of $n \geq 3$ mice.

Supplementary Figure 5. Ventricular cardiomyocyte proliferation and apoptosis at E13.5.

Cardiomyocyte proliferation was assessed using Ki67 immunohistochemistry in *Bcar1$^{+/+}$* controls (A) versus *Bcar1^{SM22KO}* mutants (B). Apoptosis was assessed using immunohistochemistry for the apoptotic cell marker cleaved-caspase-3 in *Bcar1$^{+/+}$* controls (C) versus *Bcar1^{SM22KO}* mutants (D). Scale bars are 250µm.

Supplementary Figure 6. Animations of AMIRA false coloured rendering of outflow tract (OFT) septation at E14.5 in a *Bcar1$^{+/+}$* control compared to a littermate *Bcar1^{SM22KO}* mutant.

Animation of the length of the outflow tracts at E14.5 in a *Bcar1$^{+/+}$* control (A) compared to a littermate *Bcar1^{SM22KO}* mutant (B), showing complete failure of OFT

septation in the *Bcar1*^{SM22KO} mutant and aneurysmal dilatation of the aortic sac which has failed to remodel into the aortic and pulmonary channels. False coloured renderings of the tissue sections are highlighting the OFT (blue), heart tissue (red), and the aortic sac (purple).

Supplementary Figure 7. Pharyngeal arch artery development in *Bcar1*^{SM22KO} mutants.

At E10.5 the third, fourth and sixth pharyngeal arch arteries could be visualised by ink injections in both the *Bcar1*$^{+/+}$ controls (A, B) and the *Bcar1*^{SM22KO} mutants (C, D). LS, left side; RS, right side. Scale bars are 0.4mm.

Alpha-SMA immunostaining highlighting the pharyngeal arch arteries in the *Bcar1*$^{+/+}$ controls (E) and the *Bcar1*^{SM22KO} mutants (F). (G) A significant increase in the diameter of the pharyngeal arch arteries of the *Bcar1*^{SM22KO} mutants compared to *Bcar1*$^{+/+}$ controls was detected (p=0.03 as determined by 2-tailed t-test, *Bcar1*$^{+/+}$ n=3, *Bcar1*^{SM22KO} n=3). Scale bars are 500µm.

At E13.5 the pharyngeal arch arteries had remodelled in the *Bcar1*$^{+/+}$ controls (H), whereas severe defects in vascular remodelling were observed in the *Bcar1*^{SM22KO} mutant (I), with a single outflow tract and an abnormally dilated aortic sac apparent. Abbreviations: LCA – left carotid artery ; RCA – right carotid artery ; LSA – left subclavian artery ; RSA – right subclavian artery; OFT – outflow tract; AS – aortic sac. Scale bars are 1.6mm.

Supplementary Figure 8. Cell proliferation and cardiomyocyte migration in *Bcar1*^{SM22KO} OFT cushions.

High rates of proliferation were seen in the OFT cushion mesenchyme in the *Bcar1*^{SM22KO} mutants (B), whereas in control hearts a zone of non-proliferating, quiescent cells could be clearly seen in the aortico-pulmonary complex region (asterisk in A). Apoptosis in cells of the OFT cushions was determined using Cleaved Caspase-3 IHC (C, D). MF20 staining, which specifically labels cardiomyocytes, highlights the extent of cardiomyocyte invasion during myocardialisation of the OFT in the *Bcar1*^{SM22KO} embryos (F,H) compared to littermate controls (E,G) at E13.5. (I) A significant reduction in cardiomyocyte migration was seen in the *Bcar1*^{SM22KO} mutants compared to *Bcar1*$^{+/+}$ controls ($p=0.039$ as determined by 2-tailed t-test, *Bcar1*$^{+/+}$ n=3, *Bcar1*^{SM22KO} n=4) (I). (A,B,D) scale bars are 200µm; (C, E-H) scale bars are 400µm.

Supplementary Figure 9. Expression of proteins involved in cytoskeletal remodelling and cell migration.

Phalloidin is a marker of F-actin fibres. Actin polymerisation appears to be negatively affected in the knockout, with reduced F-actin staining (phalloidin) seen in the cushion tissue in the knockout embryos (A) compared to controls (B). Cdc42 expression in control hearts (C,D) was largely limited to the OFT cushions which had fused to form the aortico-pulmonary complex. This was similar to the expression seen in the *Bcar1*^{SM22KO} embryos (E,F) where Cdc42 was also largely confined to the cells of the OFT cushions. (D,F) are magnified views of boxed regions in (C,E) respectively. (A,B) scale bars are 50µm; (C,E) scale bars are 200µm; (D,F) scale

bars are 80µm. Western blot analysis of Bcar1, Talin, Fak and Rac1 protein expression in E11.5 OFT protein lysates (G). (H) Besides a significant reduction in Bcar1 protein levels, no significant change in Talin, Fak or Rac1 expression levels was detected in protein lysates from *Bcar1*^{SM22KO} mutants compared to *Bcar1*$^{+/+}$ controls (*Bcar1*$^{+/+}$ n=6, *Bcar1*^{SM22KO} n=4, **p=0.0008 as determined by 2-tailed t-test).

Supplementary Figure 10. Mendelian ratios and phenotype of *Bcar1*^{PAX3KO} mutants.

(A) Second heart field– specific knockout of Bcar1 results in viable *Bcar1*^{MEF2CKO} mice that are born at the expected Mendelian ratio.

(B) Neural crest cell – specific knockout of Bcar1 by Pax3-Cre results in embryonic lethality by E15.5.

At E12.5 *Bcar1*^{PAX3KO} mutant embryos (H) showed similar development of the ventricles as their littermate controls (C), however, the proximal OFT failed to septate (J) compared to littermate controls (E). The distal OFT did divide into the aortic and pulmonary channels, however an abnormally dilated, unremodelled, aortic sac could be seen joining up to the aorta in the *Bcar1*^{PAX3KO} mutants (L). Asterisk indicates the aorticopulmonary septum. (C,D,E,H,I,J) scale bars are 500µm, (F,G,K,L) scale bars are 250µm.

Supplementary Figure 11. AMIRA false coloured animations of outflow tract (OFT) septation at E12.5 in a *Bcar1*$^{+/+}$ control compared to a littermate *Bcar1*^{PAX3KO} mutant.

Animation of the length of the outflow tract in a *Bcar1*$^{+/+}$ control (A) compared to a littermate *Bcar1*^{PAX3KO} mutant (B) at E12.5, showing defective OFT septation in the *Bcar1*^{PAX3KO} mutant and aneurysmal dilatation of the aortic sac. False coloured renderings of the tissue sections are highlighting the OFT (blue), heart tissue (red), and the aortic sac (purple).

Supplementary Figure 1

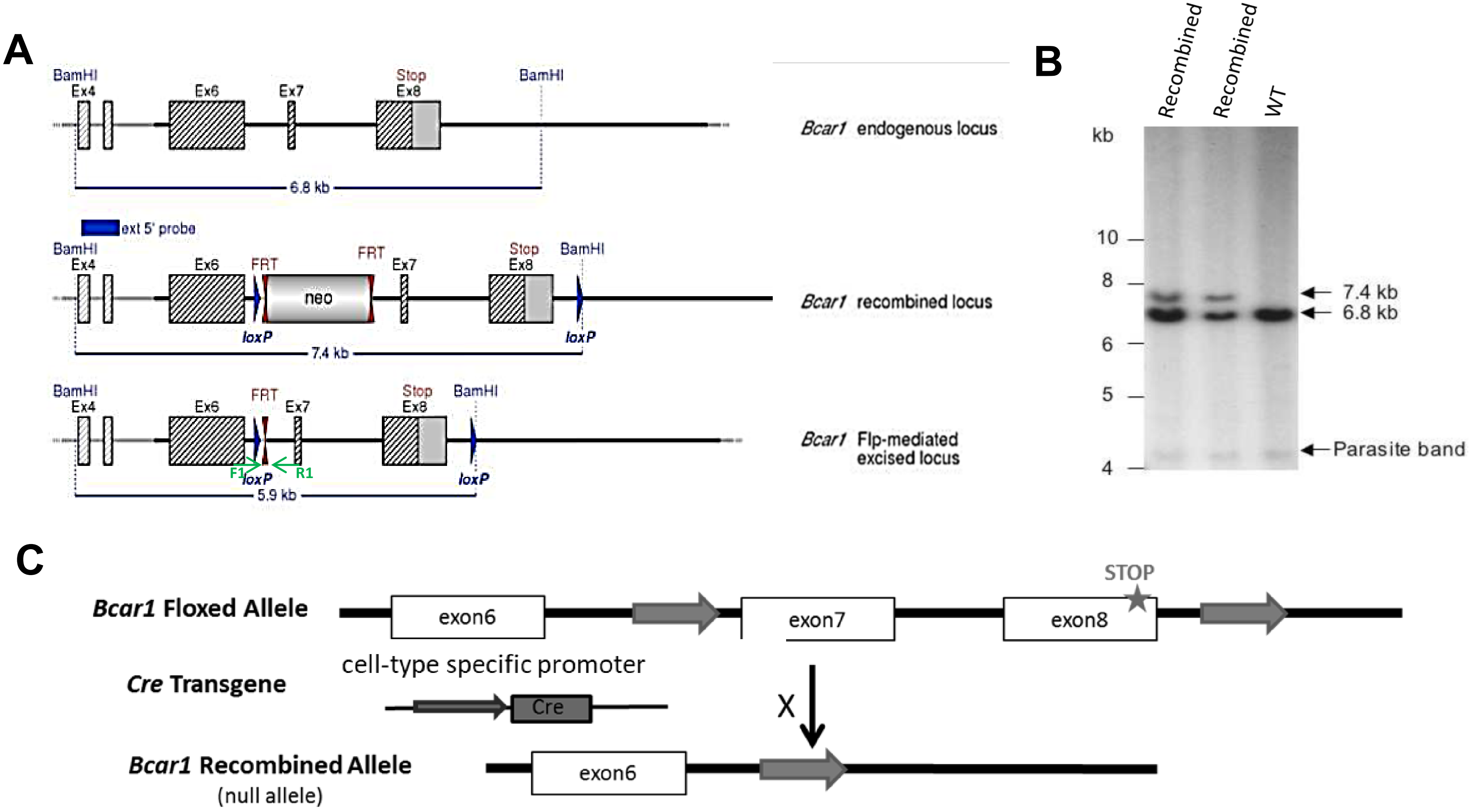

Supplementary Figure 2

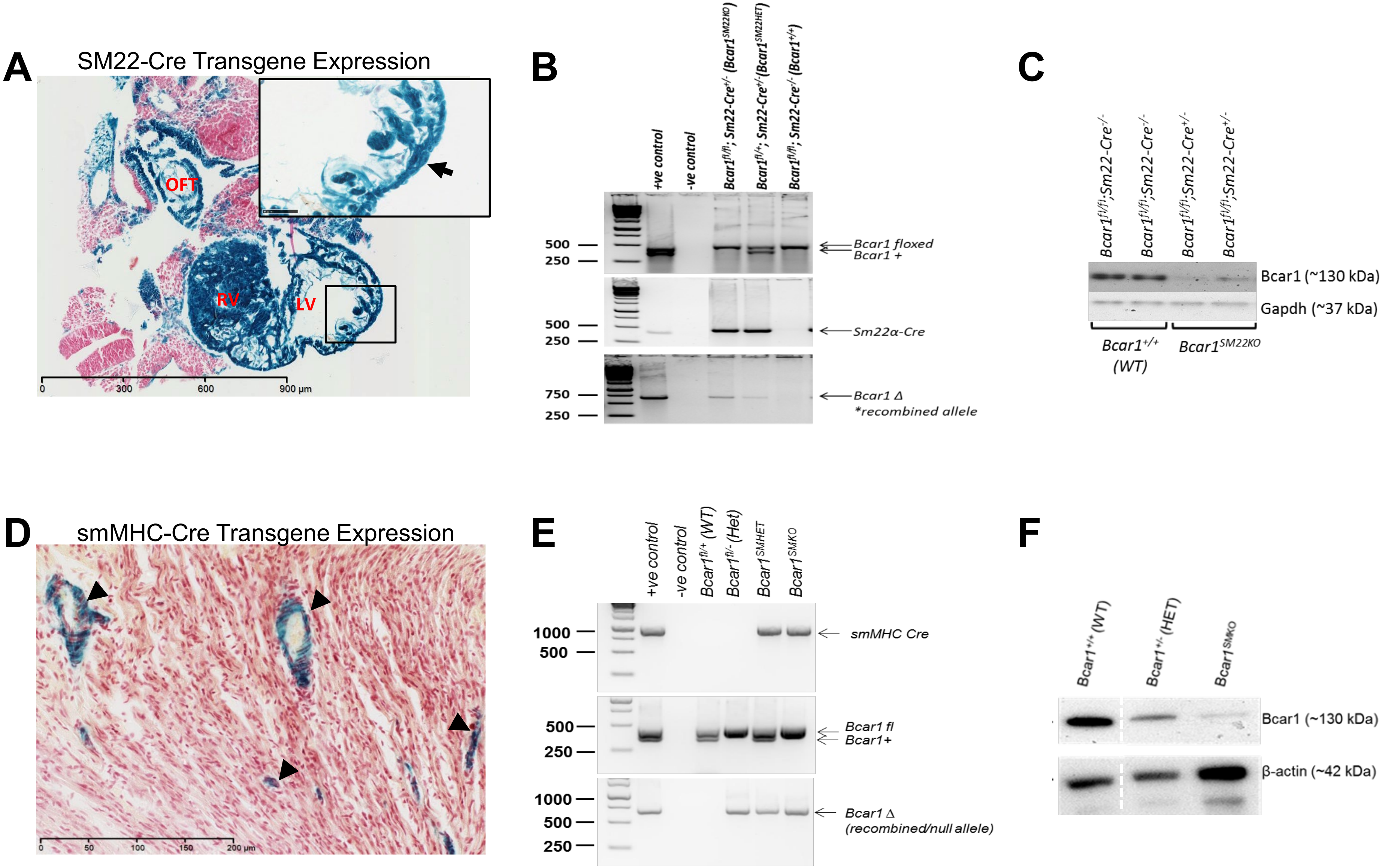

Supplementary Figure 3

A

SM22-Cre$^{+/-}$; Bcar1 $^{fl/+}$ X *Bcar1 $^{fl/+}$/Bcar1 $^{fl/fl}$*

***Bcar1* Genotype**	**Mendelian Ratio**	**Expected**	**Observed**
Bcar1$^{+/+}$ (Bcar1$^{+/+}$, SM22-Cre$^{+/-}$; Bcar1$^{+/+}$, SM22-Cre$^{-/-}$; Bcar1$^{fl/fl}$, SM22-Cre$^{-/-}$; Bcar1$^{fl/+}$, SM22-Cre$^{-/-}$)	57%	102.125	129
Bcar1^{SM22HET} (Bcar1$^{fl/+}$, SM22-Cre$^{+/-}$)	25%	45	51
Bcar1^{SM22KO} (Bcar1$^{fl/fl}$, SM22-Cre$^{+/-}$)	18%	32.875	0
**Total:**	**100%**	**180**	**180**

C

smMHC-Cre$^{+/-}$ Bcar1$^{fl/+}$ X *Bcar1$^{fl/fl}$*

Tie2-Cre$^{+/-}$ Bcar1$^{fl/+}$ X *Bcar1$^{fl/+}$*

***Bcar1* Genotype**	**Expected Mendelian Ratio (*smMHC-Cre* line)**	**Observed (*smMHC-Cre* line)**	**Expected Mendelian Ratio (*Tie2-Cre* line)**	**Observed (*Tie2-Cre* line)**
Wild-type	50%	57% (20)	62.5%	42.9% (12)
Bcar1$^{fl/+}$; Cre$^{+/-}$ heterozygote	25%	20% (7)	25%	46.4% (13)
Bcar1$^{fl/fl}$; Cre$^{+/-}$ knockout	25%	23% (8)	12.5%	10.7% (3)
Total	100%	100% (35)	100%	100% (28)

B

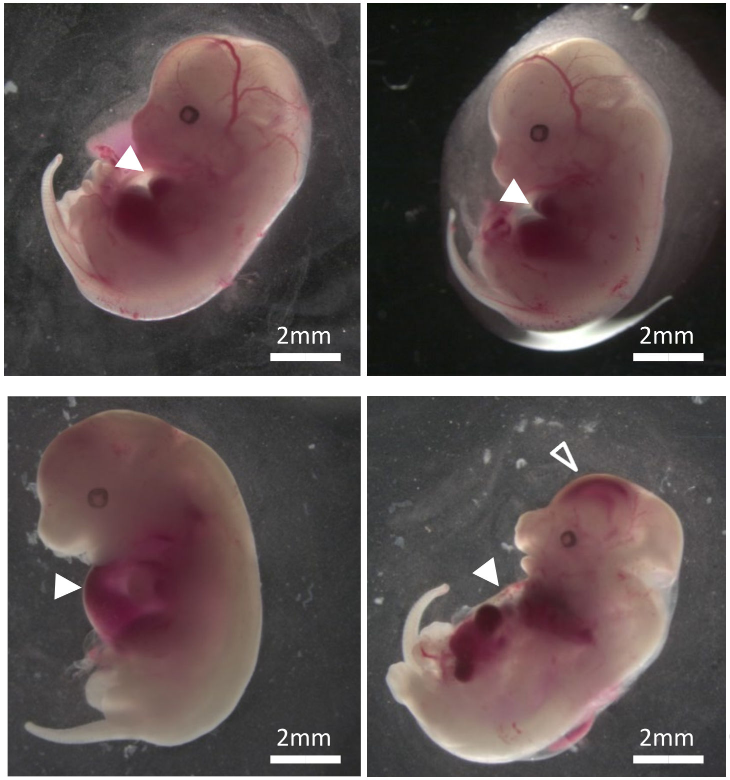

D

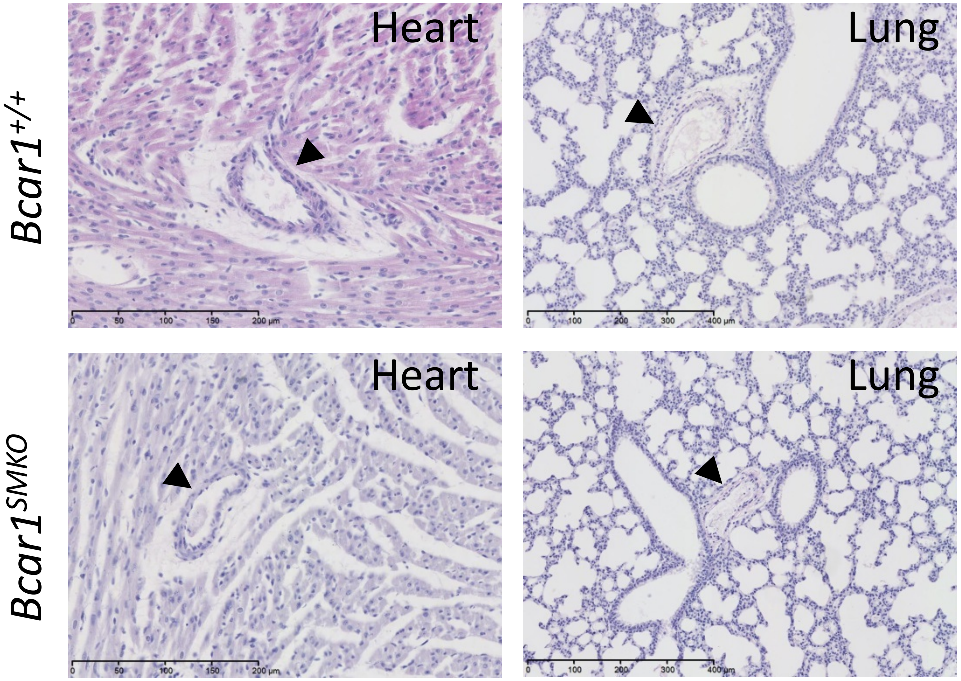

CVR-2021-0393R1

Supplementary Figure 4

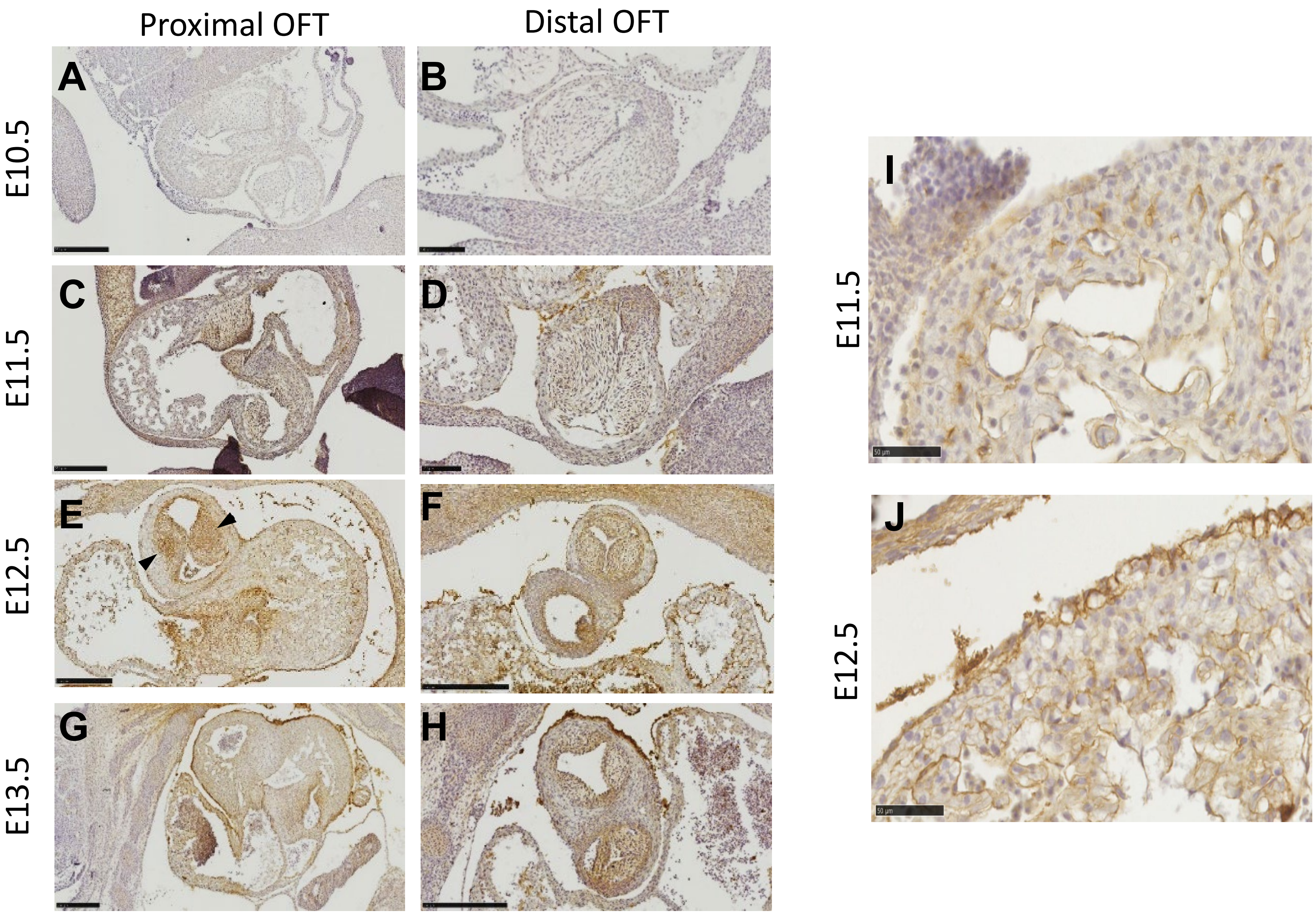

Supplementary Figure 5

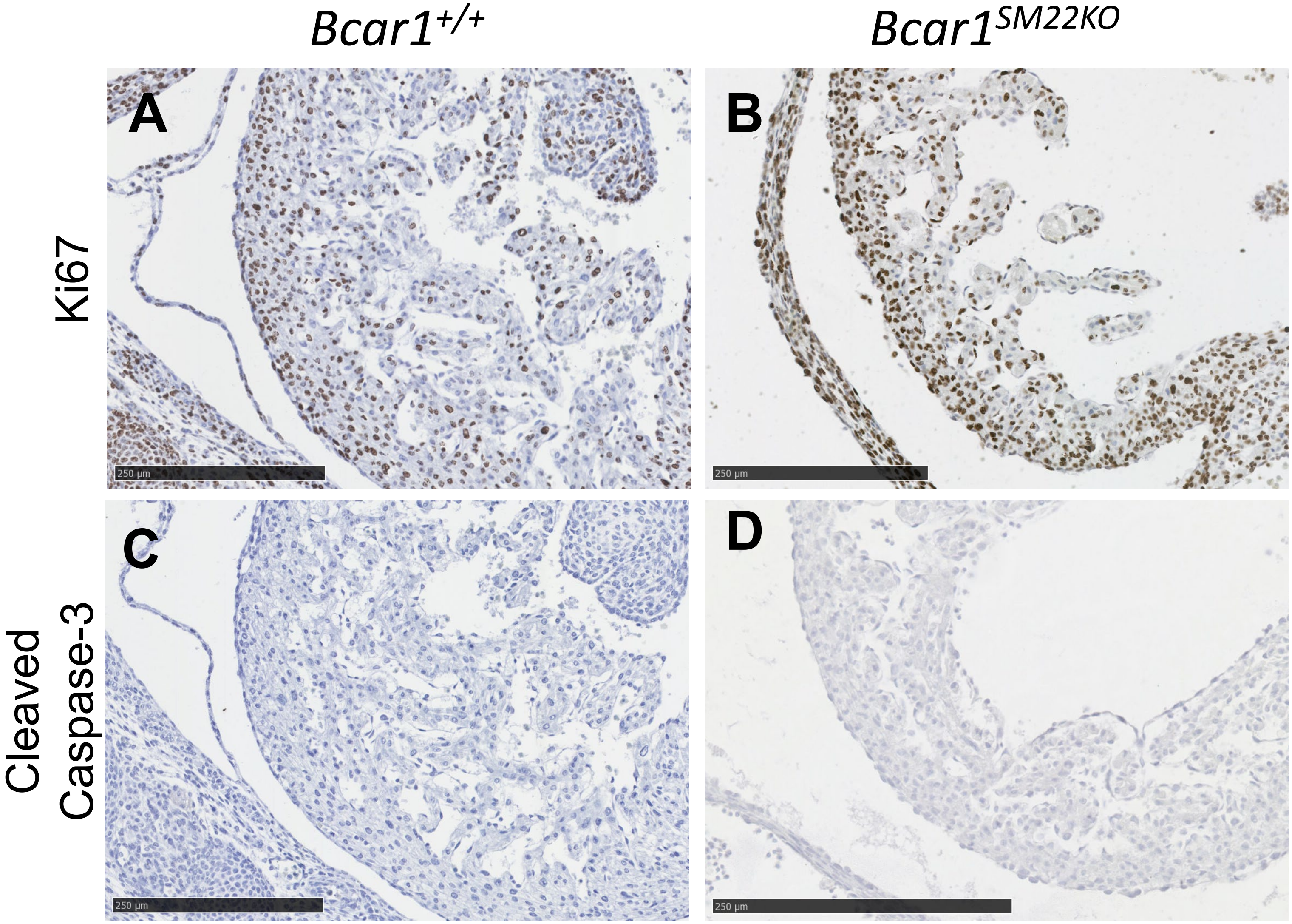

Supplementary Figure 6

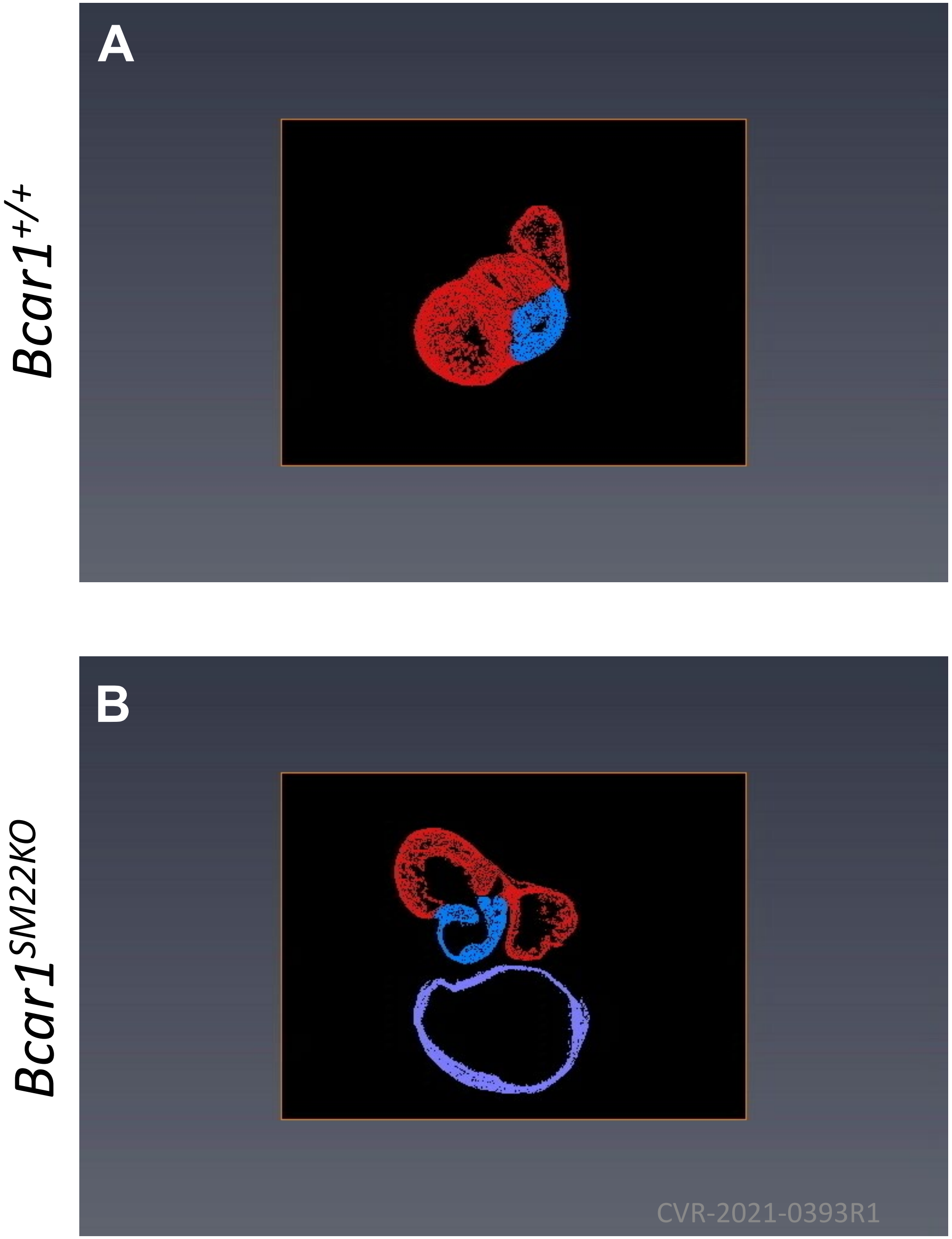

Supplementary Figure 7

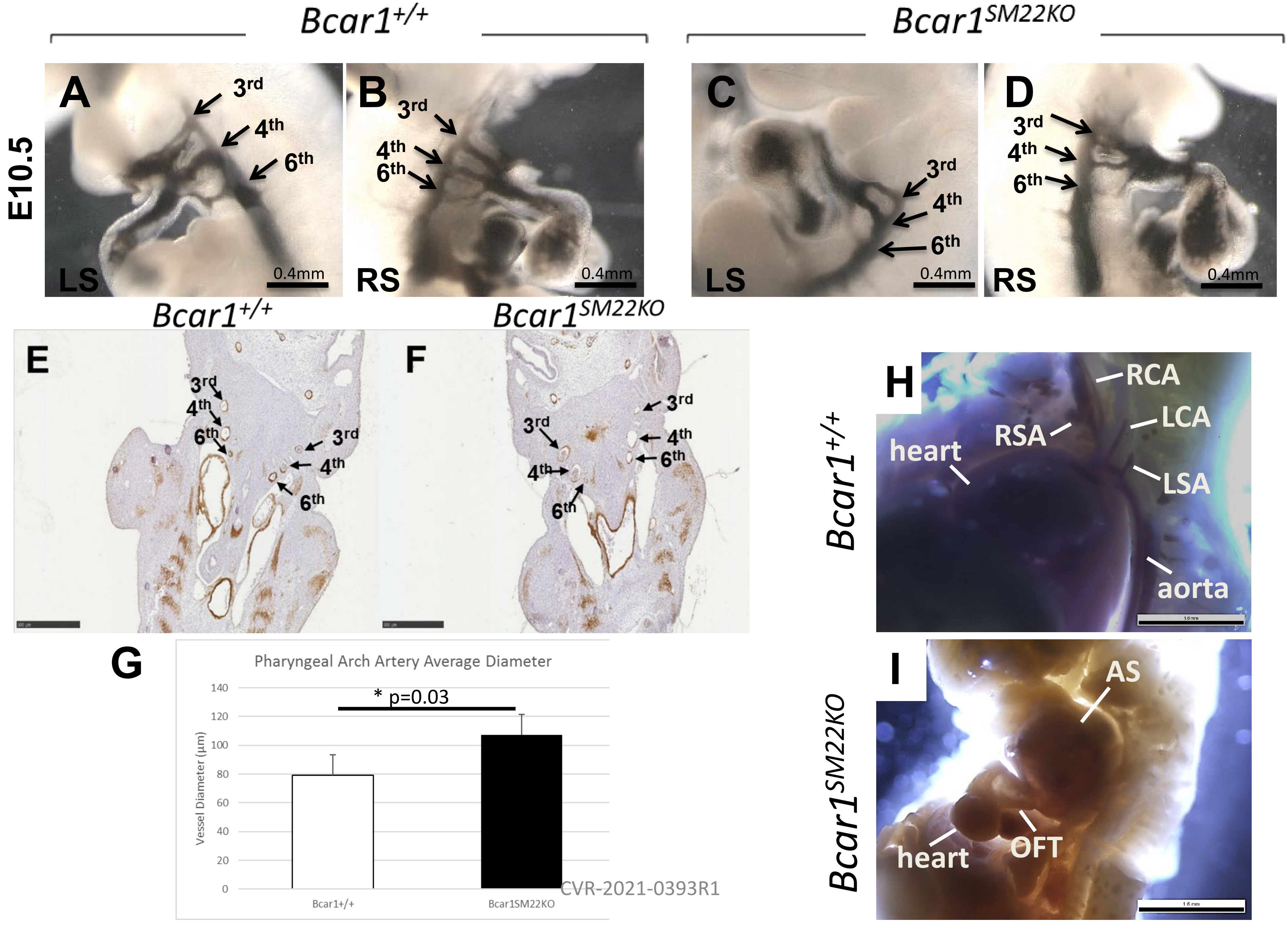

CVR-2021-0393R1

Supplementary Figure 8

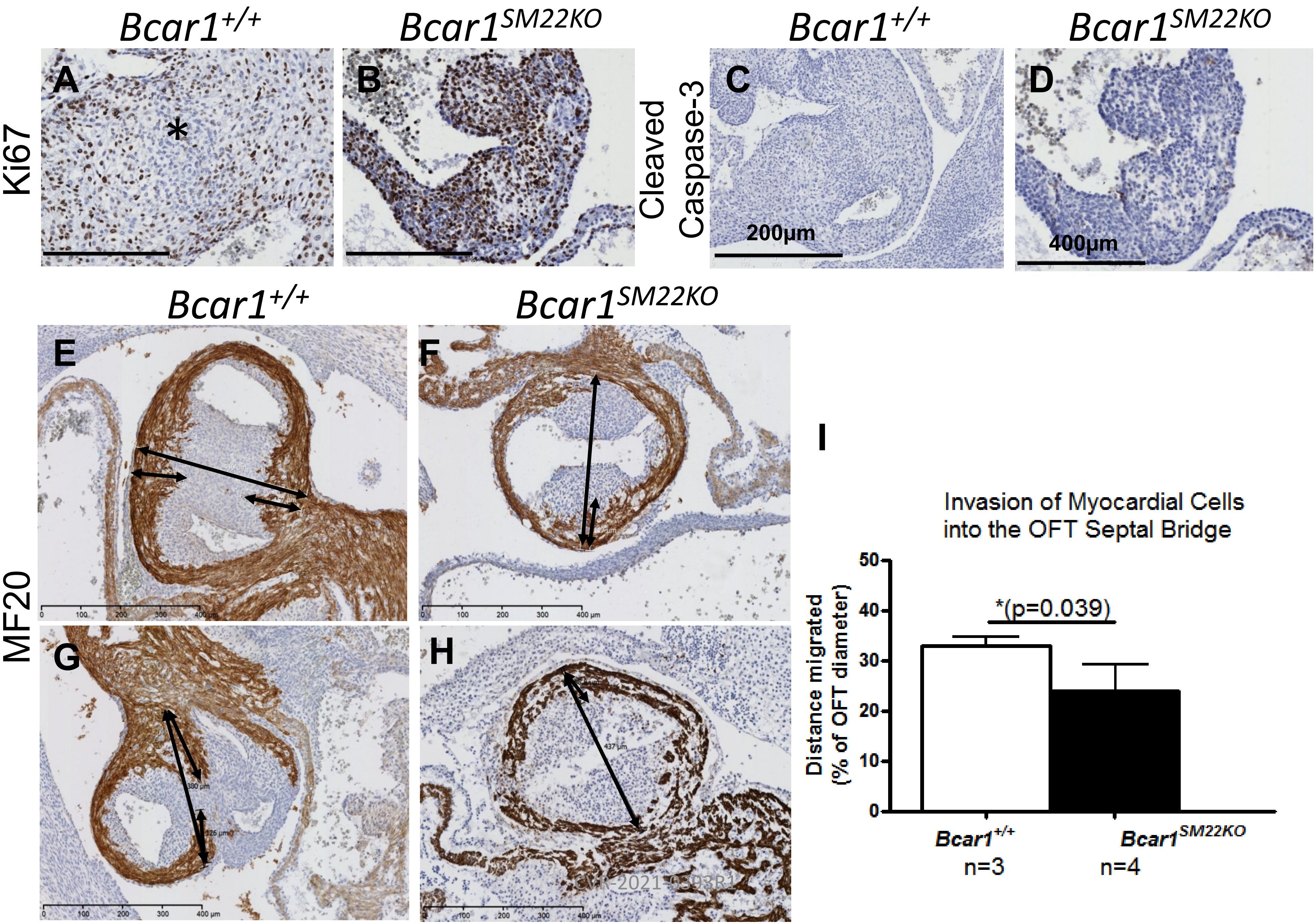

CVR-2021-0393R1

Supplementary Figure 9

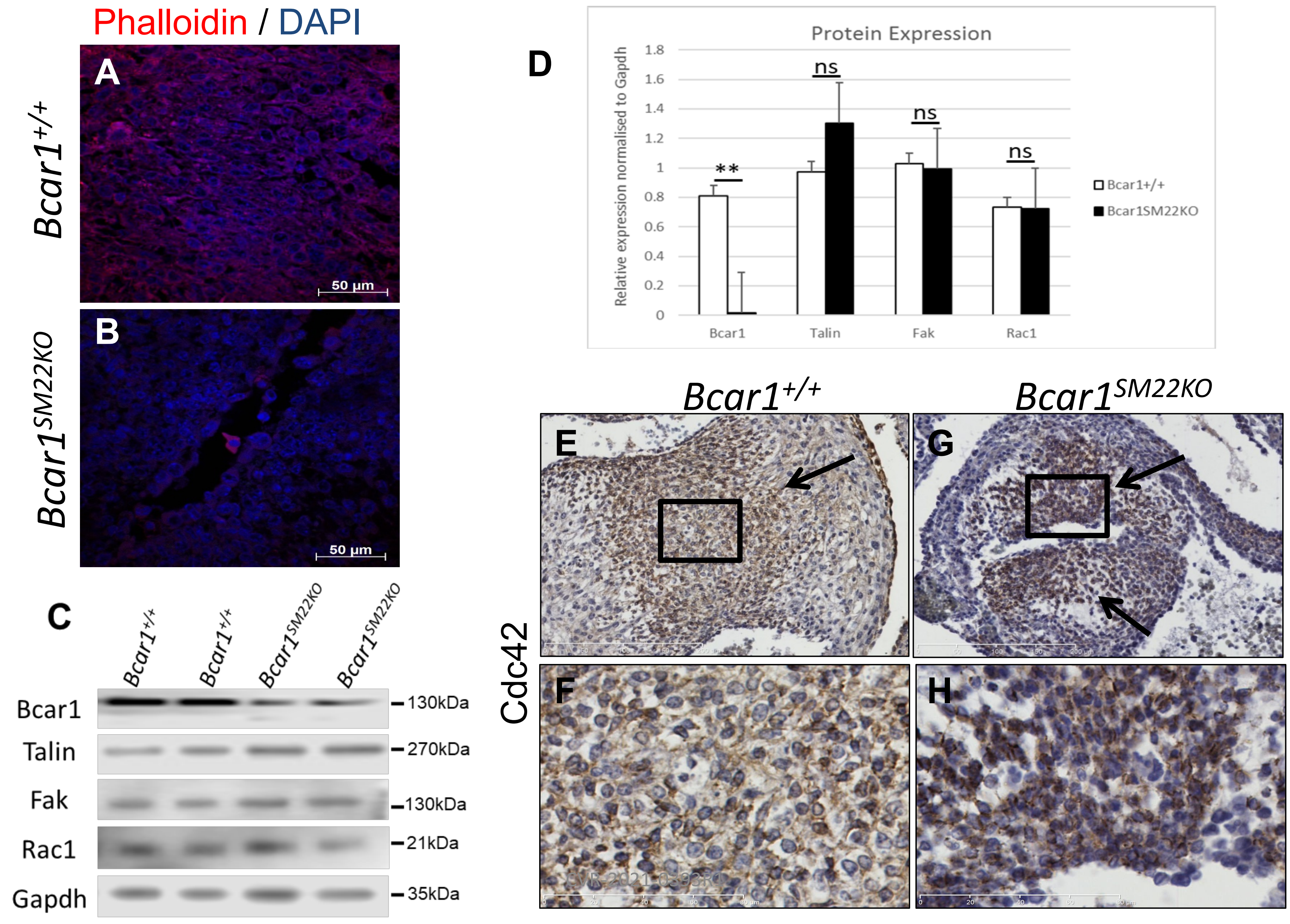

Supplementary Figure 10

A

Bcar1/p130Cas Genotype	Mendelian Ratio	Expected	Observed
Bcar1$^{+/+}$	50%	26	30
Bcar1$^{MEF2C\text{-}HET}$	25%	13	10
Bcar1$^{MEF2C\text{-}KO}$	25%	13	12
Total:	100%	52	52

B

Bcar1/p130Cas Genotype	Mendelian Ratio	Expected	Observed
Bcar1$^{+/+}$	50%	17.5	26
Bcar1$^{PAX3\text{-}HET}$	25%	8.75	9
Bcar1$^{PAX3\text{-}KO}$	25%	8.75	0
Total:	100%	35	35

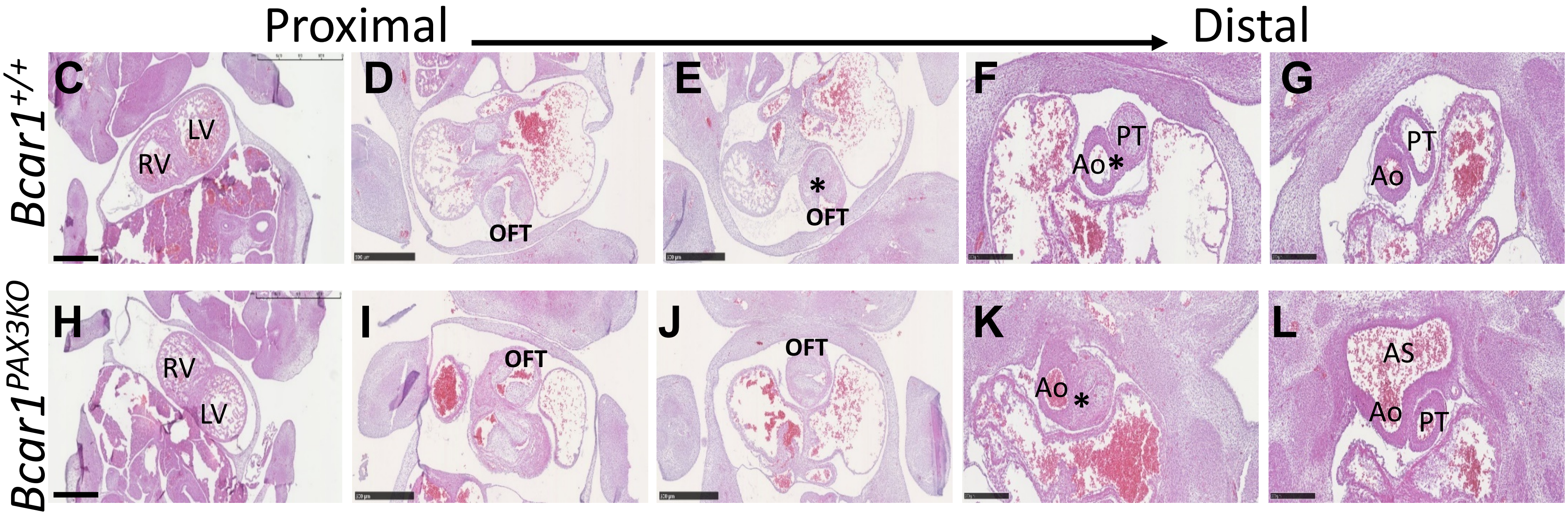

Supplementary Figure 11

$Bcar1^{+/+}$

$Bcar1^{PAX3KO}$

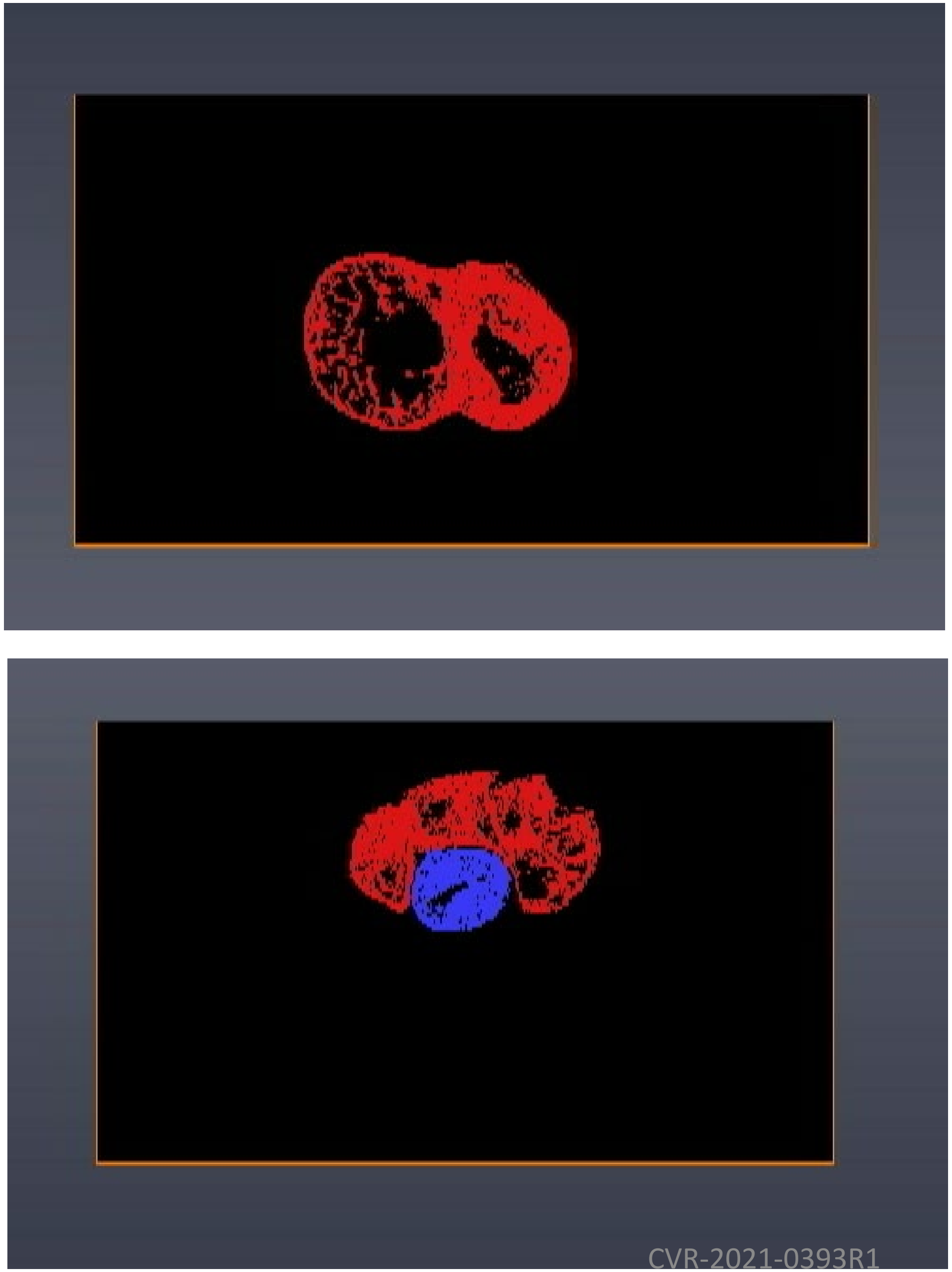

CVR-2021-0393R1
