# Supplementary figures and images for: Bcar1/p130Cas is essential for ventricular development and neural crest cell remodelling of the cardiac outflow tract
Source: Cardiovasc Res. 2021 Jul 16;118(8):1993–2005. doi: 10.1093/cvr/cvab242 (PMC9239580; doi:10.1093/cvr/cvab242)

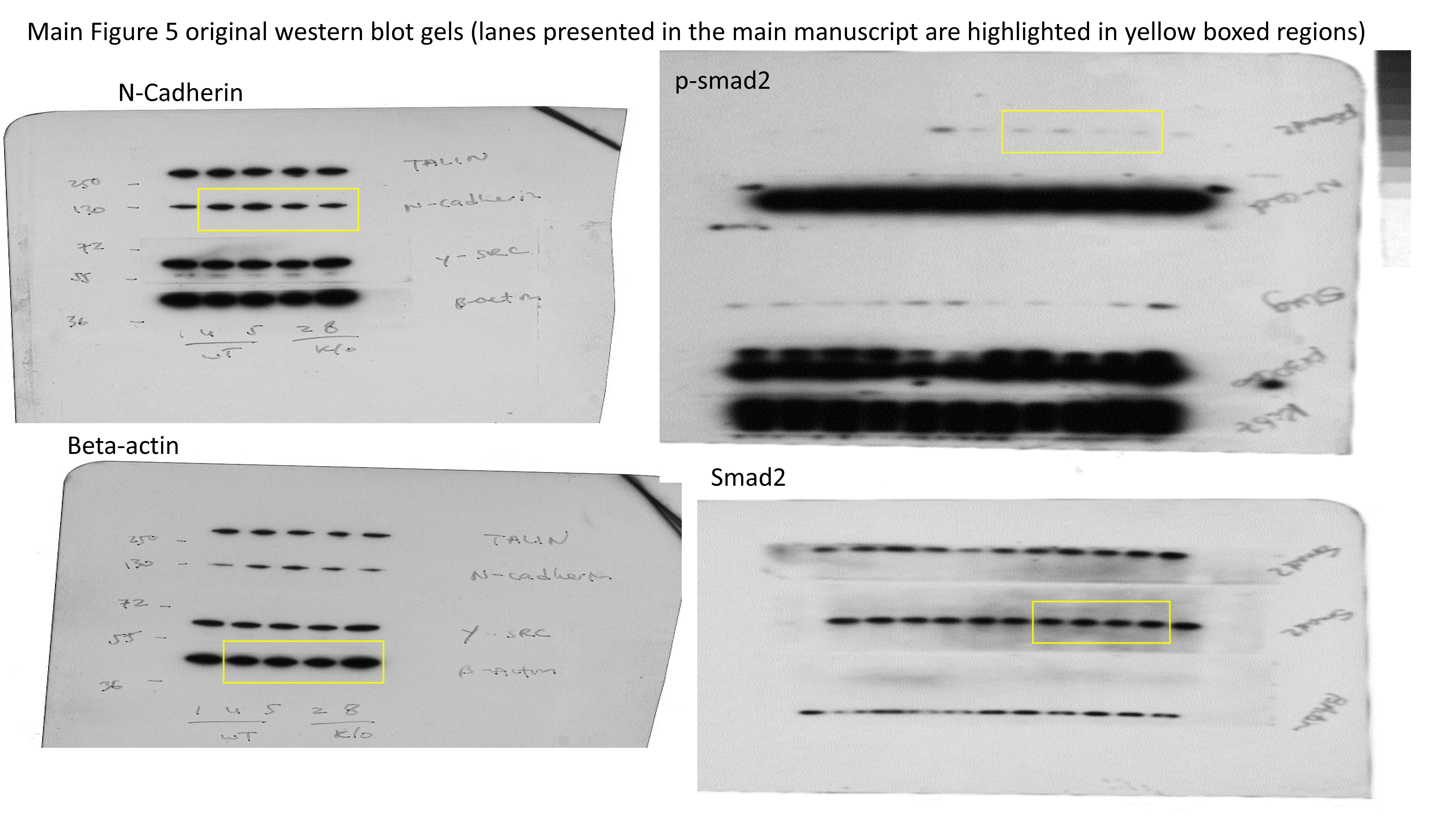

Supplement: cvab242_Supplementary_Data [file cvab242_supplementary_data.zip › unedited western blot gels_Fig5.tiff]

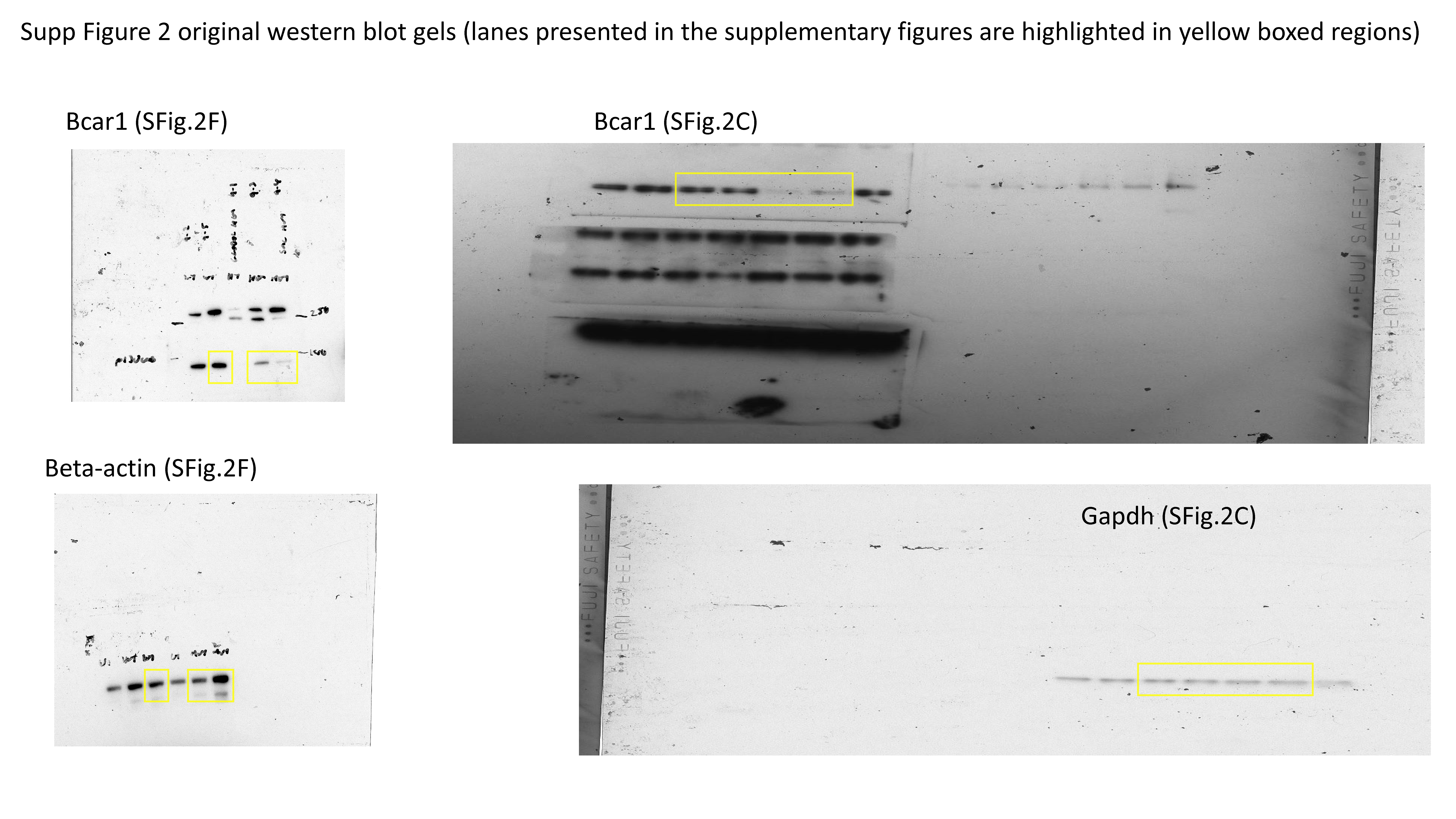

Supplement: cvab242_Supplementary_Data [file cvab242_supplementary_data.zip › unedited western blot gels_SFig2.tiff]

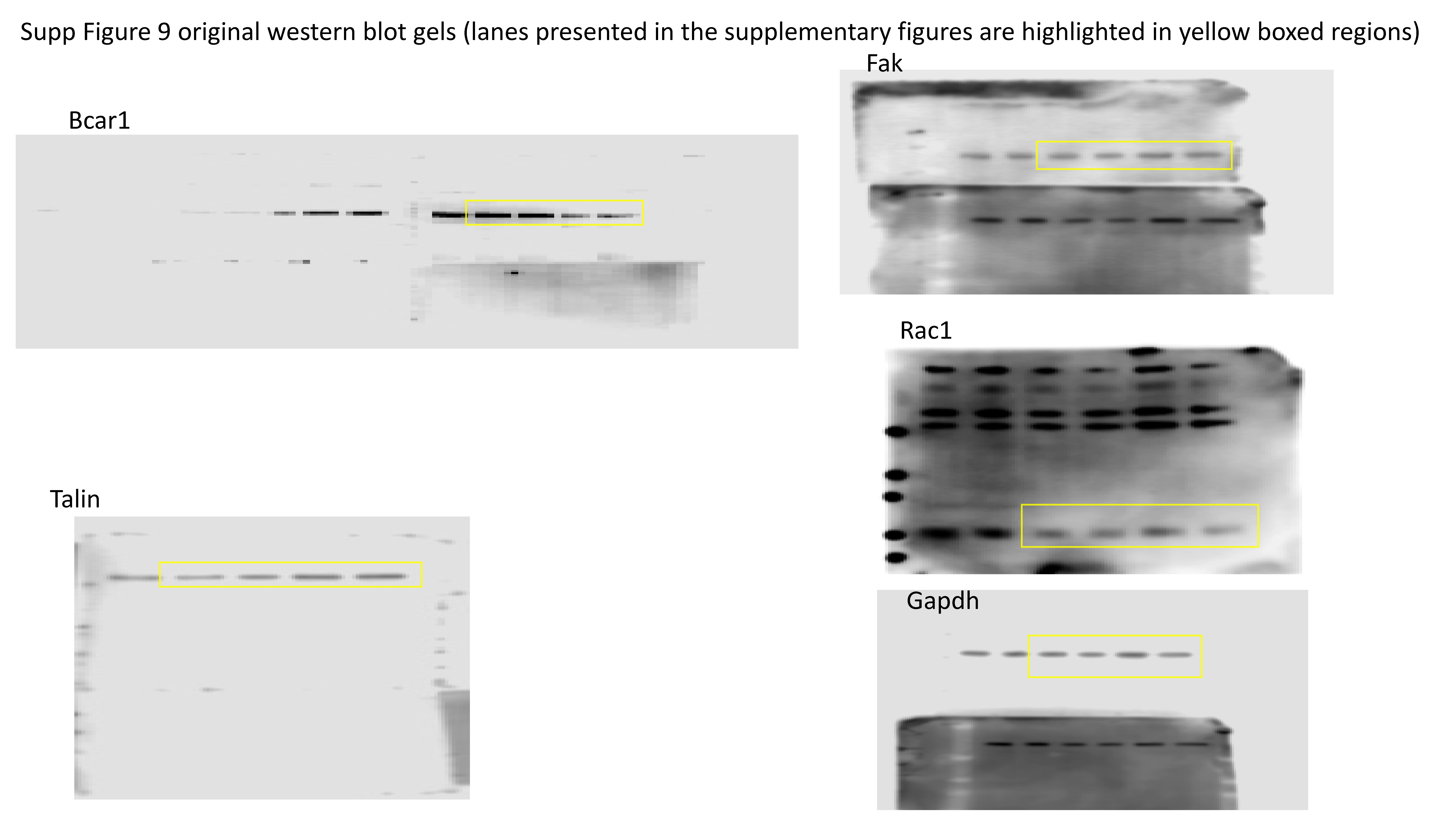

Supplement: cvab242_Supplementary_Data [file cvab242_supplementary_data.zip › unedited western blot gels_SFig9.tiff]
